# Going beyond the mean in examining relationships of adolescent non-cognitive skills with health-related quality of life and biomarkers in later-life

**DOI:** 10.1016/j.ehb.2020.100923

**Published:** 2020-12

**Authors:** Rose Atkins, Alex James Turner, Tarani Chandola, Matt Sutton

**Affiliations:** a Health Organisation, Policy and Economics, University of Manchester, Manchester, UK; b Cathie Marsh Institute for Social Research, University of Manchester, Manchester, UK; c Melbourne Institute: Applied Economic and Social Research, University of Melbourne, Melbourne, Australia

**Keywords:** Non-cognitive skills, Health-related quality of life, Biomarkers, Unconditional quantile regression

## Abstract

Several studies have established associations between early-life non-cognitive skills and later-life health and health behaviours. However, no study addresses the more important policy concern about how this relationship varies along the health distribution. We use unconditional quantile regression to analyse the effects of adolescent non-cognitive skills across the distributions of the health-related quality of life at age 50 and biomarkers at age 45 years. We examine the effects of measures of conscientiousness, agreeableness and neuroticism recorded at age 16 for 3585 individuals from the National Child Development Study. Adolescent conscientiousness is positively associated with ability to cope with stress and negatively associated with risk of cardiovascular disease in middle-age. Adolescent agreeableness is associated with higher health-related quality of life and lower physiological ‘wear and tear’, but negatively associated with ability to cope with stress in middle-age. Adolescent neuroticism is associated with lower health-related quality of life, higher physiological ‘wear and tear’, and a higher risk of cardiovascular disease in middle-age. All of these associations are stronger at the lower end of the health distribution except for the cardiovascular risk biomarkers. These associations are robust to correcting for attrition using inverse probability weighting and consistent with causal bounds assuming proportional selection on observables and unobservables. They suggest policies that improve non-cognitive skills in adolescence could offer most long-term health benefit to those with the poorest health.

## Highlights

• We examined the association between adolescent non-cognitive skills and adult health, using both subjective and objective measures of health.
• Conscientiousness is positively associated with your ability to cope with stress and negatively associated with cardiovascular risk.
• Agreeableness is positively associated with health-related quality of life and negatively associated with physiological ‘wear and tear’, however is negatively associated with your ability to cope with stress.
• Neuroticism is negatively associated with health-related quality of life, and positively associated with physiological ‘wear and tear’ and cardiovascular risk.
• All of these associations are stronger at the end of the health distribution that indicates poorer health, except for the cardiovascular risk biomarkers.

## 1 Introduction

It is widely accepted that the development of capabilities in early-life plays an important role in promoting well-being across the life course (Knudsen et al., 2006). Taking inspiration from the human capital literature, dynamic models of child development place particular importance on the role of health, cognitive skills and non-cognitive skills in shaping child development and later-life outcomes (Conti and Heckman, 2012). These multi-period models highlight how these capabilities are shaped by one another; and emphasise the importance of investing in these capabilities, particularly in early-life. However, recent evidence has begun to emphasise the long-term benefits of investment in adolescence (Bundy et al., 2018; Kirabo et al., 2020).

Of these three capabilities, the role of non-cognitive skills in determining later-life outcomes has been the least explored. Child health and cognition have long been recognised as important predictors of outcomes, such as test scores (Broman et al., 1975; Edwards and Grossman, 1979; Shakotko et al., 1981; Berkowitz and Stern, 2018), educational attainment (Wolfe, 1985; Rohde and Thompson, 2007; Grossman, 2010), labour market outcomes (Contoyannis and Dooley, 2010; Lin et al., 2016) and health (DeFries et al., 2009; Wang et al., 2018).

However, more recently there has been growing evidence that non-cognitive skills are as important as health and cognitive skills as predictors of these later-life outcomes (Borghans et al., 2008; Almlund et al., 2011; Cobb-Clark, 2015; Schurer, 2017; Gensowski, 2018; Schurer et al., 2019). In particular, non-cognitive skills are becoming increasingly recognised as important predictors of health. Associations have been found between early-life non-cognitive skills and health behaviours, such as smoking and alcohol consumption, in both adolescence and at age 30 (Conti and Heckman, 2010; Coneus and Laucht, 2014). Social maladjustment at age 11 has been shown to be associated with worse physical and mental health at ages 42 and 46 (Carneiro et al., 2007; Jones et al., 2011). Locus of control and self-esteem in childhood and adolescence have been shown to be positively associated with general health at ages 29 and 41 (Murasko, 2007; Kaestner and Callison, 2011). Furthermore, lower levels of childhood conscientiousness has been shown to predict more physiological dysregulation, greater obesity and worse lipid profiles at age 51 (Hampson et al., 2013). Non-cognitive skills have also been shown to contribute to the education-health gradient to an extent nearly as large as that of cognition (Conti and Hansman, 2013).

The current literature is limited by the lack of objective health measures used. Although there is a large body of psychology literature that looks at the association between the ‘Big Five’ and biomarkers, the majority of these studies measure both non-cognitive skills and health in adulthood (Sutin et al., 2010a; Luchetti et al., 2014; Roh et al., 2014; Elliot et al., 2017; Soliemanifar et al., 2018; Christensen et al., 2019). Biomarkers are objectively measured and evaluated as an indicator of normal biological processes, pathogenic processes, or pharmacological responses to a therapeutic intervention (Atkinson et al., 2001) and are therefore not impacted by reporting bias. Biomarkers have been shown to predict later-life disability and healthcare utilisation (Davillas and Pudney, 2019, 2020) and provide objective information on pre-disease pathways by measuring physiological processes that are beyond an individual’s perception of their own health (Jürges et al., 2013). Biomarkers have been used in studies looking at the relationship between: income and health (Davillas et al., 2017); education and health (Powdthavee, 2010; Jürges et al., 2013; Courtin et al., 2019); and socioeconomic status and health (Dowd and Goldman, 2006; Na-Ek and Demakakos, 2017). Biomarkers have also been used to study inequality of opportunity in health (Carrieri and Jones, 2018; Galárraga et al., 2018).

The current literature is also limited by its focus on the effect of non-cognitive skills on average levels of health. Focusing on the effects at the mean ignores potential effects at other parts of the health distribution, such as at its extremes (Bitler et al., 2006). This is important for policy makers as very poor health is associated with high costs to the health care system (Goetzel et al., 1998), and large welfare losses for the individual through a negative impact on employment (Carrieri and Jones, 2017). Using continuous measures of health, such as biomarkers, allows studies to go beyond the mean.

In this study, we extend the current literature by using the RIF regression approach developed by Firpo, Fortin and Lemieux (2009) to assess the relationship between non-cognitive skills and health at different points of the unconditional health distribution. The method works by applying the recentred influence function (RIF) transformation at each quantile of the outcome distribution, creating a series of binary variables for whether an observation’s outcome is above or below each quantile. Linear probability regression can then be used to estimate marginal effects of covariates at each point of the distribution. Using data from the National Child Development Study (NCDS), we use RIF regressions to examine how the association between multiple non-cognitive skills measured at age 16 differ across quantiles of the distribution of health. In doing so, we add to several recent studies that have used RIF regressions to study determinants of health (Carrieri and Jones, 2017, 2018; Davillas et al., 2017; Davillas and Jones, 2018).

We make a further contribution to the literature by recognising the multidimensionality of non-cognitive skills. Many studies use a composite measure of non-cognitive ability, implicitly treating non-cognitive skills as a single skill. Previous studies show this risks underestimating their importance (Humphries and Kosse, 2017). We draw on the ‘Big Five’ definition of non-cognitive skills: openness to experience, conscientiousness, extraversion, agreeableness and neuroticism (Almlund et al., 2011; Borghans et al., 2008). Due to data availability we are only able to create separate measures of three skills: conscientiousness, agreeableness and neuroticism.

Furthermore, we build on previous studies by examining associations between adult health and non-cognitive skills measured in adolescence. This is particularly important as there has been an identified need for more research on the effects of mental health, which can be measured using skills such as neuroticism, in the “missing middle” of adolescence (Currie, 2020).

Lastly, we are the first to study the relationship between adolescent non-cognitive skills and objective health measures. It is important not to rely solely on subjective health measures as non-cognitive skills have been shown to impact how individuals report their health (Jorm et al., 1993; de Jonge and Slaets, 2005d). This could potentially induce bias in the relationship between non-cognitive skills and true levels of health. We combine information from multiple biomarkers to create a proxy for overall physiological ‘wear and tear’ (Davillas and Jones, 2018; Le Castagné et al., 2018), as well as examining associations between non-cognitive skills and separate biomarkers of stress and cardiovascular risk.

Our results suggest adolescent non-cognitive skills are associated with later-life health, and that the magnitude and direction of these associations varies across different non-cognitive skills, health measures, and over different parts of health distribution. Previous studies estimating associations at the mean mask heterogeneity in associations across different parts of the health distribution. Associations are generally strongest in the poorer health part of the health distribution. Our results suggest that higher adolescent agreeableness is positively associated with health-related quality of life, whereas higher adolescent neuroticism is negatively associated with health-related quality of life. For both of these skills, we find that the association is larger for those reporting poorer health-related quality of life. We also find that higher adolescent agreeableness is associated with a decrease in physiological ‘wear and tear’, whereas higher adolescent neuroticism is associated with an increase in physiological ‘wear and tear’. Furthermore, we find that adolescent conscientiousness is positively associated with your ability to cope with stress, whereas adolescent agreeableness is negatively associated with your ability to cope with stress. For all of these associations, the magnitudes are largest in parts of the biomarker distributions indicative of poor health. Lastly, we find that higher adolescent conscientiousness is associated with a decreased risk of cardiovascular disease, whereas higher adolescent neuroticism is associated with an increased risk of cardiovascular disease. Unlike previous results, we find these associations to be stronger for those with lower risk of cardiovascular disease.

The remainder of this paper is organised as follows. Section 2 describes the data and section 3 presents our methodology. Our results are presented in section 4, and section 5 discusses the findings.

## 2 Data

### 2.1 The national child development study (NCDS)

We use data from the NCDS; a longitudinal study of around 17,000 children born in the UK between 3rd and 9th March 1958. The study has its origins in the “Perinatal Mortality Survey”, which was designed to examine the social and obstetric factors associated with stillbirth and death in early infancy (Brown & Hancock, 2015). This study draws on the data collected between 1958 and 1974, in addition to data collected as part of the biomedical survey when the cohort members are aged 44–45 (2002/3) and further data collected at age 50 (2008). The initial survey contains information collected from the mother and gathered from the child’s medical records. The subsequent interviews were carried out with parents, teachers, and school health services; and a range of questions were answered by the cohort members themselves.

### 2.2 Non-cognitive skills

We create indicators of three of the ‘Big Five’ non-cognitive skills: conscientiousness, agreeableness, and neuroticism. A conscientious individual is one who is organised, hardworking, responsible and willing to comply with conventional rules. Individuals that exhibit agreeableness tend to be compassionate, cooperative, altruistic and forgiving. Neuroticism can be seen as a chronic level of emotional instability, and is often associated with impulse control. A neurotic individual often exhibits emotions such as anxiety, depression, a lack of self-confidence and irritability (Almlund et al., 2011).

The ‘Big Five’ were not measured using the common International Personality Item Pool (IPIP) in the NCDS until the cohort was aged 50. Instead, we create proxy measures from the available data recorded in adolescence. We derived all three non-cognitive skills variables from teacher-reported measures of attitudes when the participants were aged 16.

Measures of neuroticism and agreeableness are derived from the Rutter Scale (Rutter, 1967). The overall Rutter Scale has been shown to have satisfactory re-test and inter-rater reliability (Rutter, 1970), and sub-scores have been shown to be efficient in differentiating children with neurotic or antisocial disorders (Rutter, 1967). The scale consists of 26 brief statements concerning the child’s behaviour, and each statement is rated: “certainly applies”, “applies somewhat” or “doesn’t apply”. These are given a weight of “2″, “1″ and “0” respectively.

We measure neuroticism using the Rutter “neurotic” sub-score (Rutter, 1967), which is obtained by summing the score of the following four items: “often worried, worries about many things”; “often appears miserable, unhappy, tearful or distressed”; “tends to be fearful or afraid of new things or new situations”; and “has tears on arrival at school or has refused to come into the building this year”. The sub-score is increasing in neuroticism.

We measure agreeableness using the Rutter “anti-social” sub-score (Rutter, 1967), which is obtained by summing the scores of the following six items: “often destroys own or others’ belongings”; “frequently fights with other children”; “is often disobedient”; “often tells lies”; “has stolen things on one or more occasions”; and “bullies other children”. This sub-score is reverse-coded to create a measure increasing in agreeableness.

There are several validated taxonomies for conscientiousness (Costa and McCrea, 1992; Chernyshenko, 2003; MacCann et al., 2009; Green et al., 2016). Despite differences, there is significant overlap in the facets included in each. All include some measure of how hardworking an individual is; represented by facets such as industriousness, perseverance, striving and achievement. They all also include a measure of self-control or self-discipline, as well as some measure of cautiousness; represented by cautiousness itself or facets such as deliberation and responsibility. Lastly, although they are not included in all of the taxonomies, facets such as perfectionism, competence, traditionalism and virtue can also be found in the definitions.

We create measures of three conscientiousness facets (hardworking, rigidity, cautiousness) from teacher-reported measures of attitudes recorded at age 16. Teachers were asked to rate the student on three scales: lazy-hardworking, flexible-rigid and impulsive-cautious. Rigidity is used as a proxy for perfectionism, given evidence that individuals who are more rigid in their thinking are also more perfectionistic (Ferrari and Mautz, 1997). The facet scales range from 1 to 5 and are increasing in conscientiousness. We then use information on the facets to create an aggregate measure of conscientiousness. This is done by coding each individual facet to range from 0 to 4 and summing to create an index running from 0 to 12.

Our measures of agreeableness and neuroticism are converted to the same 0–12 scale as the conscientiousness measure.

### 2.3 Health

#### 2.3.1 Health-related quality of life

We measure health-related quality of life at age 50 using the six-dimensional health state short form (SF-6D), which is a health-related quality of life index derived from the short-form 36 health survey (SF-36; Ferreira et al., 2013). The SF-36 yields health scores across eight dimensions: limitation in physical activities because of health problems; limitations in social activities because of physical or emotional problems; limitations in usual role activities because of physical health problems; bodily pain; general mental health (psychological distress and well-being); limitations in usual role activities because of emotional problems; vitality (energy and fatigue); and general health perception (Ware and Sherbourne, 1992). The SF-6D is a preference-based index derived from 11 items of the SF-36, which are combined into six dimensions of health: physical functioning, role limitations, social functioning, pain, mental health and vitality (Brazier et al., 2002). The SF-6D describes 18,000 different health states. A representative sample of the UK population valued these health states and econometric models were estimated to predict utility scores for all health states defined by the SF-6D. We apply these utility scores to SF-6D responses to derive the SF-6D utility index; a continuous measure ranging from 0.35 to 1.00 (Ferreira et al., 2013). A value of 1 indicates perfect health-related quality of life, and a value of 0.35 indicates the poorest health-related quality of life.

#### 2.3.2 Biomarkers

We use biomarker data collected from NCDS respondents at age 44/45 years. We follow Le Castagné et al. (2018) and Solis et al. (2015) by using information from 14 biomarkers representing four physiological systems: salivary cortisol t1 and salivary cortisol t1-t2 (neuroendocrine system); insulin-life growth factor-1, C-reactive protein, fibrinogen and Immunoglobulin E (immune and inflammatory system); high-density lipoprotein, low-density lipoprotein, triglycerides and glycated haemoglobin (metabolic system); systolic blood pressure, diastolic blood pressure, heart rate and peak expiratory flow (cardiovascular and respiratory systems). A full description of these biomarkers can be found in Appendix Table A1.

We first combine the information on all biomarkers to create a measure of allostatic load. Allostatic load is a measure of global physiological ‘wear and tear’ and centres on the brain’s stress response to environmental challenges over the life course (McEwen, 2000). To create the measure, we follow Davillas and Jones (2018) by first converting each biomarker to a z statistic before averaging the z statistics across all biomarkers. This was preferred to the approach taken in Le Castagné et al. (2018) and Solis et al. (2015) which converts each biomarker into a binary variable for high-risk levels prior to aggregation. This discards information about the distribution of these individual biomarkers. Higher levels of allostatic load indicate higher physiological ‘wear and tear’.

We also examine effects on individual biomarkers from each physiological system. These include: cortisol t1-t2, which measures stress response; triglyceride-HDL ratio, which is a metabolic biomarker for cardiovascular risk; and C-reactive protein (CRP), which is an inflammatory biomarker for cardiovascular risk.^1^ Cortisol samples in the NCDS were taken in the first 45 min after waking (t1) and 3 h later (t2). A slower decrease in cortisol levels through out the day (flatter diurnal cortisol slopes, or a smaller difference between the t1 and t2 cortisol values) is an indicator of high levels of stress and is associated with health conditions such as depression, fatigue, cardiovascular disease and type II diabetes (Adam et al., 2017; Bower et al., 2005; Doane et al., 2013; Hackett et al., 2016; Kumari et al., 2009; Matthews et al., 2006).

Mild increases in CRP levels (2–10 mg/L) is an indicator of metabolic inflammation and is associated with chronic diseases such as cardiovascular disease and type II diabetes (Brenner et al., 2014; Yale et al., 2016). High levels of triglyceride (>200 mg/dL) is shown in patients with incident ischemic stroke, and is associated with increased cardiovascular risk (Miller et al., 2011; Upadhyay, 2015; Wedro, 2018). HDL is a type of cholesterol, and is commonly known as the “good” cholesterol as high levels are associated with reduced levels of cardiovascular disease (Castelli et al., 1986; Fisher et al., 2012). Therefore, the higher the triglyceride-HDL ratio, the greater the risk of cardiovascular disease. Due to the skewness of the biomarker distributions, they are log-transformed prior to analysis. Biomarker distributions are depicted in Appendix Fig. A3.

### 2.4 Covariates

We derive a set of covariates based on those used in Jones, Rice and Dias (2012). We control for childhood health using a morbidity measure that aggregates 13 categories of health conditions affecting a child at age 7. The morbidity index is an aggregate of points, where one point is attributed to the occurrence of each of the following conditions: infectious diseases; ear and throat problems; recurrent acute illnesses; acute illnesses (other); asthma; bronchitis and wheezing; allergies; chronic diseases (medical); chronic physical or mental handicap; chronic sensory illnesses; injuries; psychological problems; psychosomatic problems; other childhood morbidity (unspecified). In addition, we include parental health variables to enable us to account for hereditary conditions.^2^

Parental background characteristics were measured using mothers’ and fathers’ years of schooling, mothers’ and fasters’ age (in quadratic form), sex of the cohort member, parity of mother in 1958 (measuring the number of previous pregnancies), and region of residence in 1958. We also control for household financial difficulties during the cohort member’s childhood and adolescence, mothers’ and fathers’ country of birth, an interaction term between mothers’ and fathers’ age, and fathers’ (or mothers’ husbands’) social class.

We additionally control for cognitive ability in childhood, using test scores measured at age 11. These comprise tests in maths, reading, general ability verbal, general ability non-verbal and copying designs. We combine the two general ability test scores into a single measure, leaving us with four variables measuring different dimensions of cognitive ability. Further information on the covariates is provided in Table 1.

**Table 1:** Descriptive statistics for the non-cognitive skills and the covariates.

Variable	Mean	Std. Dev.
Conscientiousness	6.45	1.67
Agreeableness	11.55	1.33
Neuroticism	1.07	1.62
Test scores (age 11)		
General ability	47.29	14.96
Reading	17.28	5.90
Maths	18.88	10.14
Copying designs	8.50	1.36
Child Morbidity Index (age 11)	1.47	1.32
Mothers' Year of Birth	1930.3	5.48
Fathers' Years of Birth	1927.4	6.12
Parity of the Mother in 1958	1.18	1.36
Mothers' Years of Schooling	15.05	1.51
Fathers' Years of Schooling	15.10	1.92

Fathers’ social class		
I (Professional) – omitted category	0.05	0.22
II (Managerial and Technical)	0.16	0.36
III (Skilled – Non-Manual)	0.10	0.30
III (Skilled – Manual)	0.51	0.50
IV (Partly Skilled)	0.11	0.32
V (Unskilled)	0.06	0.24
Financial Difficulties (age 11)	0.07	0.25
Mothers' Birthplace		
England – omitted category	0.86	0.34
Wales	0.06	0.25
Scotland	0.02	0.12
Northern Ireland	0.01	0.08
Ireland	0.02	0.13
Other	0.03	0.18

Fathers' Birthplace		
England – omitted category	0.84	0.36
Wales	0.07	0.25
Scotland	0.02	0.14
Northern Ireland	0.01	0.09
Ireland	0.02	0.15
Other	0.04	0.19
Mother has a diagnosed illness	0.05	0.22
Fathers' has a diagnosed illness	0.07	0.25

Region of Residence 1958		
North	0.09	0.28
North West	0.12	0.33
Riding	0.10	0.30
North Midlands	0.10	0.30
Midlands	0.11	0.31
East	0.10	0.30
South	0.07	0.25
South East – omitted category	0.19	0.39
South West	0.08	0.26
Wales	0.06	0.24

**Notes:** N = 3,584. Figures are recorded to two decimal places. Non-cognitive skills are teacher reported and measured at age 16, with a range of 0–12.

### 2.5 Sample construction

**Fig. 1:**
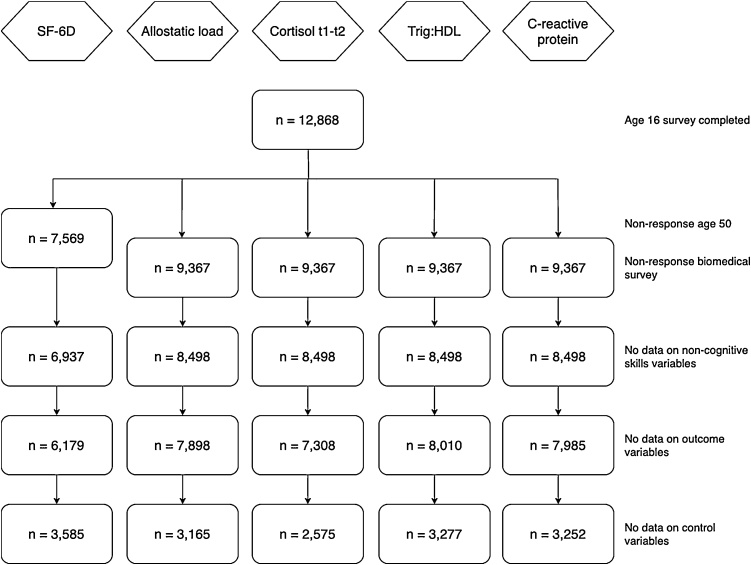
Flowchart describing the derivation of the estimation sample for each outcome. **Notes:** n = 12,868 represents those observations for which the age 11 and PMS surveys were completed prior to the completion of the age 16 survey.

Due to attrition in the NCDS and differences in missingness between outcomes, we create separate estimation samples for each outcome.^3^ Fig. 1 depicts the process of deriving these samples.

Similarly to many other studies that use the NCDS, the sizes of our final estimation samples are significantly affected by attrition (Case et al., 2005; Cutler and Lleras-Muney, 2010; Jones et al., 2011, 2012). However, several studies have analysed the implications of non-random attrition and have found that this is not a serious source of bias for models using the NCDS (Case et al., 2005; Lindeboom et al., 2009). Details on how we test for this specifically in this study are provided in Section 3.1.

## 3 Methods

We first use ordinary least squares (OLS) to examine the association between adolescent non-cognitive skills and adult health at the mean of the health distribution. Assuming no unobserved confounding, OLS regressions provide consistent estimates of the impact of an explanatory variable, X, on the *population unconditional mean* of an outcome variable Y.

We then use the Recentred influence function (RIF) method to examine the association between adolescent non-cognitive skills and adult health, along the unconditional health distributions (Firpo et al., 2009). For the $\tau^{th}$ quantile, the RIF can be written as:

$$RIF\left(y;q_{\tau }\right)=q_{\tau }+\;\frac{\tau -\;I\{y\leq q_{\tau }\}}{f_{Y}(q_{\tau })}$$

where $q_{\tau }$ is the unconditional quantile, $I\left\{y\leq q_{\tau }\right\}$ is an indicator variable equal to one for observations with an outcome value less than or equal to the observed quantile and zero otherwise, and $f_{Y}\left(q_{\tau }\right)$ is the density of the outcome at the $\tau^{th}$ quantile.

After estimating (${\hat{q}}_{\tau }$) using the sample data, and the densities ($\hat{f_{Y}}\left(\hat{q_{\tau }}\right)$) using kernel density methods, the unconditional quantile partial effects, $UQPE\left(\tau \right)$, are estimated through OLS regression of the estimated RIFs on the set of covariates.

The unconditional quantile of the outcome variable, $q_{\tau },$ can be obtained as follows:

$$q_{\tau }=\;E_{x}[E\left[\hat{RIF}\left(Y,q_{\tau }\right),X\right]]$$

Where $\hat{RIF}\left(Y,q_{\tau }\right)|X$ is the estimate of the RIF , conditional on covariates X. As a result of this linear approximation, it is possible to apply the law of iterated expectations:

$$q_{\tau }=E[X]{\hat{\delta }}_{\tau }$$

Where ${\hat{\delta }}_{\tau }$ is the coefficient of the unconditional quantile regression. This linearization allows estimation of the marginal effect of a change in distribution of the covariates (including non-cognitive skills) on the unconditional outcome quantiles measured by the parameters $\;{\hat{\delta }}_{\tau }$ (Carrieri and Jones, 2017).

We apply the RIF approach with all measures of adolescent non-cognitive skills included in the same model (conscientiousness, agreeableness, and neuroticism), to estimate the association between each adolescent non-cognitive skill and health at 5th, 10th, 25th, 50th, 75th, 90th and 95th quantiles of the distributions of biomarkers and health-related quality of life, controlling for the set of covariates outlined in Section 2.4.^4^

All non-cognitive skills are converted to z statistics prior to analysis, such that coefficient represent associations between adult health and one standard deviation increase in non-cognitive skills. To obtain standard errors, we bootstrap all the RIF regressions as suggested by Rios-Avila (2019).

### 3.1 Robustness checks

#### 3.1.1 Omitted variable bias

Although we control for a range of child and family background characteristics, the lack of data on some covariates (such as parental non-cognitive skills) means there is a potential for omitted variable bias. To test for the potential impact omitted variable bias on our results, we implement robustness check under the assumption that bias from observed covariates is informative about bias from unobserved covariates (Altonji et al., 2005). We implement the method proposed by Oster (2019) which uses information on the movements in both the coefficients and the R-squared values when controls are added to estimate bias-adjusted effects and causal bounds.

In the context of this study, the method considers the following data generated process:

$$RIF\left(y;\;q_{\tau }\right)={\;\alpha }_{i,\;\tau }+\;{\beta_{i,\;j,\;\tau }NCS}_{i,\;j,\;\tau \;}+\;\gamma_{i,\;\tau }X_{i,\;\tau }+\;\delta_{i,\;\tau }U_{i,\;\tau }+\;\varepsilon_{i,\;\tau }\;$$

where the subscript i stands for individual, the subscript τ stands for the quantile, and the subscript j=1, 2, 3 represents the three non-cognitive skills variables. y represents adult health and NCS represent adolescent non-cognitive skills. X is a vector of observed control variables, and U is a vector of all potential unobserved confounders.

The estimate of $\beta_{\tau }$ from this impossible regression represents the causal effect of an increase in adolescent non-cognitive skills on adult health, at quantile τ. $R_{\tau }^{max}$ denotes the R-squared from this regression, at quantile τ. Performing an uncontrolled regression, removing both $X_{i,\;\tau }$ and $U_{i,\;\tau },$ would result in the biased coefficient estimate, ${\ddot{\beta }}_{\tau },$ and an R-squared, ${\ddot{R}}_{\tau },$ at quantile τ. Regressing the RIF of our outcomes on ${NCS}_{i,\;j,\;\tau \;}$ and $X_{i,\;\tau }$ (the baseline specification), results in the less biased estimate, ${\tilde{\beta }}_{\tau },$ and a R-squared, ${\tilde{R}}_{\tau },$ at quantile τ.

Oster (2019) suggests that a bias-adjusted effect (Eq. (1)), $\beta_{\tau }^{*}$ can be estimated as follows:

(1) $$\beta^{\text{*}}\left(R^{max},\;q_{\tau },\;\delta \right)=\;{\tilde{\beta }}_{\tau }-\left[\delta \left({\ddot{\beta }}_{\tau }-\;{\tilde{\beta }}_{\tau }\right)\frac{R_{\tau }^{max}-\;{\tilde{R}}_{\tau }}{{\tilde{R}}_{\tau }-\;{\ddot{R}}_{\tau }}\right]$$

δ denotes degree of selection on unobservables, which in this study is the strength of the relationship between ${NCS}_{i,\;j,\;\tau \;}$ and the unobservables, $U_{i,\;\tau },$ relative to the strength of the relationship between ${NCS}_{i,\;j,\;\tau \;}$ and the observed covariates, $X_{i,\;\tau }.\;\delta$ is assumed >0 and is $<1\left(>1\right)$ if ${NCS}_{i,\;j,\;\tau \;}$ is less (more) influenced by unobservables than observables. Oster (2019) shows that $\beta^{*}$ converges to the probability of the true causal effect.

δ and $R_{\tau }^{max}$ are unobservables, and therefore assumptions must be made about their values. Oster (2019) states that, due to measurement error in the outcomes, $R_{\tau }^{max}$ is very unlikely to be close to 1, and recommends estimating $R_{\tau }^{max}$ as ${1.3\tilde{R}}_{\tau }$ (130 % of the R-squared from the baseline specification). The author also suggests that it is unlikely that δ>1. Conditional on these assumptions, Oster (2019) suggests that the causal effect will lie within the bounds (Eq. (2)):

(2) $${[\tilde{\beta }}_{\tau },\;\beta_{\tau }^{\text{*}}\left(\text{min}\left[{1.3\tilde{R}}_{\tau },\;1\right],\;1\right)]$$

Narrow causal bounds provide evidence against omitted variable bias. Also, conditional on finding a positive or negative relationship between adolescent non-cognitive skills and adult health, along the unconditional distribution of health, causal bounds not including zero provides evidence in support of a causal relationship in the same direction.

#### 3.1.2 Non-random attrition bias

Given the substantial amount of attrition between the adolescent and adult waves of the NCDS, there is a potential for sample selection to impact results. To examine this, we use inverse probability weighting (IPW) to correct RIF results for potential non-random attrition bias, under the missing at random (MAR) assumption i.e. the are no unobserved predictors of missingness other than the covariates included in our model (Seaman and White, 2013; Bhaskaran and Smeeth, 2014). IPW re-weights the sample based on the probability of attrition such that characteristics of individuals who remain in the sample are identical, on average, to the full sample. Higher weights are placed on individuals who remain in the estimation samples who are more similar in characteristics to those who are lost. This results in a sample that is representative of both those lost observations and those individuals in our original sample (Seaman and White, 2013).

To implement IPW, we first use logistic regression to regress a binary indicator for inclusion in each estimation sample on non-cognitive skills variables and control variables:

$$E_{i,s}=\;\tau_{0,s}+\;\tau_{j,\;s}{NCS}_{i,j,\;s\;}+\;\tau_{4}X_{i,\;s\;}+\;\varepsilon_{i,\;s}$$

where subscript s represents the five health outcomes, and $\;E_{i,s}=1$ if individual *i* is in the estimation sample for outcome s. From this equation we then calculate the predicted probability of being in each estimation sample (the propensity score), $\rho_{s}=pr(E_{i,s}=1|X_{i})$, given the covariates. We then, for each RIF regression, weight observations by the inverse of the propensity score.

## 4 Results

### 4.1 Descriptive statistics

#### 4.1.1 Non-cognitive skills

**Fig. A1:**
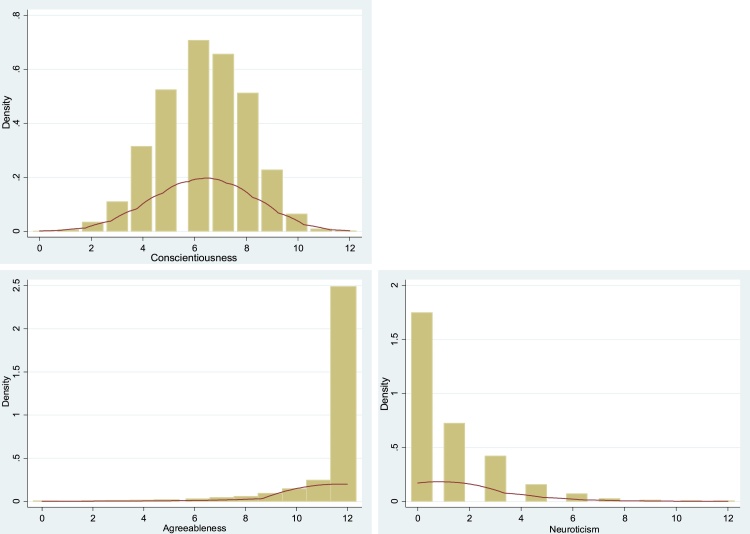
Distributions of teacher-reported conscientiousness, agreeableness, and neuroticism at age 16.

Distributions of each adolescent non-cognitive skill are depicted in Appendix Fig. A1. Adolescent conscientiousness is approximately normally distributed, with a mean close to the midpoint of the outcome range. In comparison, the mean for adolescent agreeableness lies at the upper end of the range and the distribution is heavily skewed to the right, suggesting cohort members have high levels of adolescent agreeableness on average. Conversely, the mean for adolescent neuroticism lies at the lower end of the range and the distribution is heavily skewed to the left, suggesting cohort members have low levels of adolescent neuroticism on average.

#### 4.1.2 Covariates

Table 1 shows descriptive statistics for the covariates. We use test scores as proxies for cognitive ability. General ability has a mean of 47.29 and a range of 0–79, reading has a mean of 17.28 and a range of 0–35, maths has a mean of 18.88 and a range of 0–40, and copying designs has a mean of 8.50 and a range of 0–12.

Average years of schooling is similar between fathers and mothers. On average, mothers had one full term pregnancy before 1958. Only 7 % of parents reported their family having experienced financial difficulties when the cohort member was age 11. 51 % of fathers reported their social class as “III manual”. Around 85 % of mothers and fathers were born in England. 5 % of mothers and 7 % of fathers reported having an illness by the time of the age 11 survey.

#### 4.1.3 Health-related quality of life and biomarkers

**Fig. A2:**
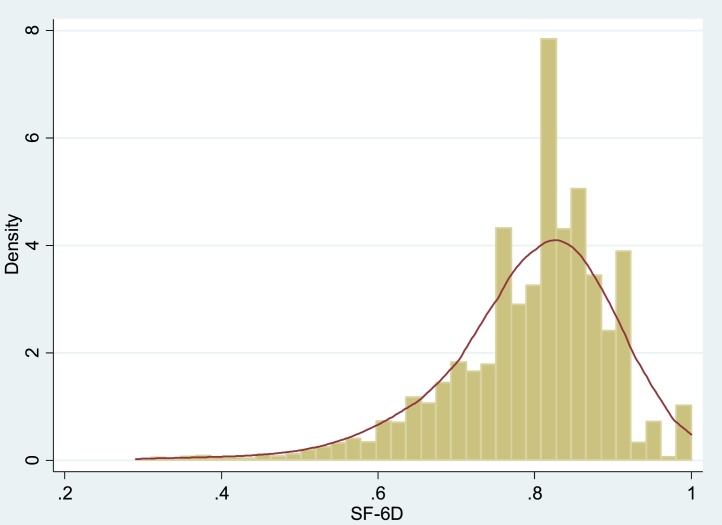
Distribution of self-reported health-related quality of life (SF-6D) measured at age 50.

**Fig. A3:**
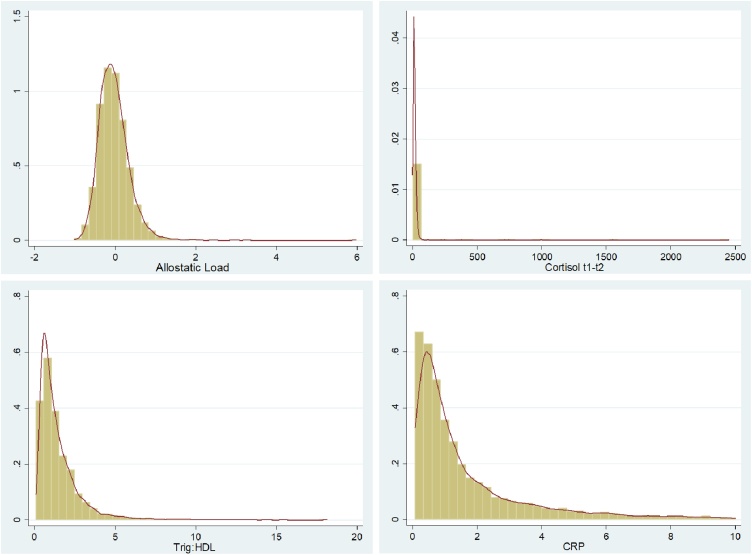
Distribution of biomarkers (allostatic load, cortisol t1-t2, triglyceride-HDL ratio and C - reactive protein (CRP)) measured at age 45.

Summary statistics for the health outcomes are provided in Table 2, and their distributions are depicted in Appendix Fig. A2, Fig. A3. The mean for the SF-6D utility index is 0.8 and its distribution is skewed towards perfect health (Appendix Fig. A2). This suggests that on average the cohort members report good levels of health-related quality of life.

**Table 2:** Descriptive statistics for the SF-6D utility index and the biomarkers.

Variable	Mean	Std. Dev.	N
SF-6D	0.80	0.10	3584
Allostatic load	−0.05	0.36	3165
Cortisol t1-t2 (nmol/L)	15.07	23.21	2575
Triglyceride-HDL ratio	1.46	1.34	3277
C-reactive protein (CRP; mg/L)	2.10	4.61	3252

**Notes:** Figures are recorded to two decimal places. Health-related quality of life (SF-6D utility index) is measured at age 50 and range between 0.35 (worst health) to 1 (perfect health). All biomarkers are measured at age 44–45.

Allostatic load has a mean around zero, and the distribution is skewed to the lower end (Appendix Fig. A3). The mean for cortisol t1-t2 is 15.07 nmol/L, with a large standard deviation of 23.21 nmol/L and a heavily left skewed distribution. Triglyceride-HDL ratio has a mean of 1.46, indicating that on average the cohort members have one and a half the amount of triglyceride compared to HDL. The closer to one this score is, the healthier the individual. The triglyceride-HDL ratio distribution is skewed to the left. CRP has a mean of 2.10 mg/l and a leftward skewed distribution, indicating that on average the cohort have healthy levels of the inflammatory biomarker.

### 4.2 Regression results

#### 4.2.1 Health-related quality of life

**Fig. 2:**
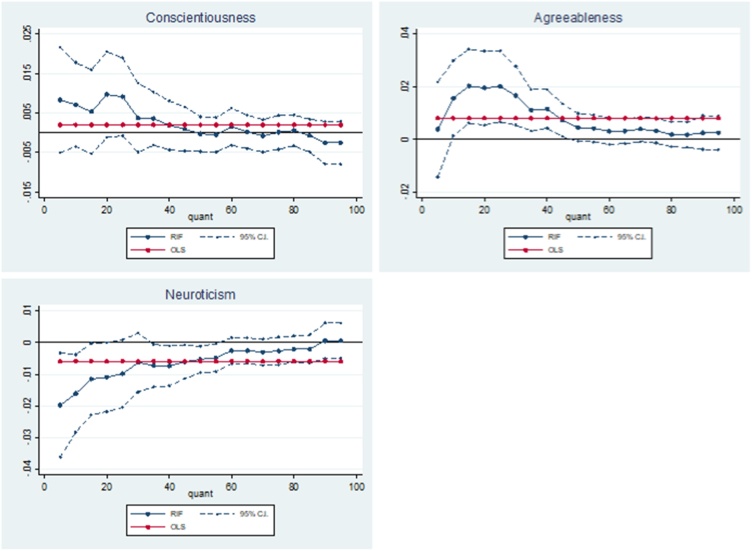
Adolescent non-cognitive skills – SF-6D relationship.

Table 3 presents OLS results alongside the results of RIF regressions which describe the association between adolescent non-cognitive skills and the unconditional distribution of the SF-6D utility index at the 5th, 10th, 25th, 50th, 75th and 90th quantile. Fig. 2 plots the relationship between the adolescent non-cognitive skills variables and the SF-6D over the whole SF-6D utility distribution. Results are presented at every 5th quantile.

**Table 3:** OLS and RIF estimates of the associations between adolescent non-cognitive skills and the SF-6D utility index.

		RIF					
	OLS	Q5	Q10	Q25	Q50	Q75	Q90
Conscientiousness	0.002	0.008	0.007	0.009*	0.000	0.000	−0.003
	(0.002)	(0.007)	(0.005)	(0.005)	(0.002)	(0.002)	(0.003)

Agreeableness	0.008***	0.004	0.016***	0.020***	0.005*	0.003	0.003
	(0.003)	(0.009)	(0.007)	(0.007)	(0.003)	(0.002)	(0.003)

Neuroticism	−0.006**	−0.020***	−0.016***	−0.010**	−0.005**	−0.003	0.001
	(0.003)	(0.008)	(0.006)	(0.005)	(0.002)	(0.002)	(0.003)

Observations	3584	3584	3584	3584	3584	3584	3584

Robust standard errors calculated from bootstrapping in parentheses: * p < 0.10, ** p < 0.05, *** p < 0.010.

**Notes:** the non-cognitive skills variables have been converted to their z statistic. The SF-6D utility index is a continuous measure ranging from 0.35 to 1.00 (Ferreira *et al.*, 2013). The results can be interpreted as a one standard deviation increase in the non-cognitive skills. Additional covariates include: age 11 test scores (general ability, reading, maths and copying design), child morbidity index, sex of the cohort member, mothers’ and fathers’ years of schooling, quadratic in mothers’ and fathers’ age in 1958, region of residence in 1958, parity of the mother in 1958, whether the mother or father had a long-term illness when the cohort member was a child, mothers’ and fathers’ birthplace, fathers’ social class and an indication of whether the parents had faced financial difficulties measured when the cohort member was 11.

Table 3 and Fig. 2 show that for all adolescent non-cognitive skills, the relationship with health varies depending on the quantile of the SF-6D distribution. Overall, the effects of each adolescent non-cognitive skill tends to be greatest at the lowest part of the health distribution, which indicates worse health-related quality of life. These results suggest that the effect at the mean is only able to explain a fragment of the relationship between adolescent non-cognitive skills and later-life health.

The OLS results show no significant positive association between adolescent conscientiousness and the SF-6D at the mean. However, the RIF results show some evidence of a significant positive relationship at the 25th quantile (0.009; p < 0.1). Although the associations are similar in magnitude to this at the lower part of the SF-6D distribution, these do not become statistically significant because the associations become more imprecisely measured. This is evident from the fact that the 95 % confidence interval for the association is greatest at the lowest end of the SF-6D distribution (Fig. 2).

The OLS results show that a one standard deviation increase in adolescent agreeableness is associated with an increase in the SF-6D at the mean (0.008; p < 0.01). The RIF results show that a one standard deviation increase in adolescent agreeableness is associated with a statistically significant increase in the SF-6D at the 10th-50th quantiles. The magnitude of the association peaks at the 25th quantile (0.020; p < 0.01) and decreases in magnitude at the higher end of the distribution, representing good health-related quality of life. As with conscientiousness, the standard errors are greatest at the lower end of the SF-6D distribution and explains why significance is lost at the 5th quantile (Fig. 2), although this is also driven by the fall in the magnitude of the association. These results suggest that adolescent agreeableness has a greater association with health-related quality of life at age 50 for those at the lower end of the unconditional SF-6D distribution.

Lastly, the OLS results show that a one standard deviation increase in adolescent neuroticism is associated with a decrease in the SF-6D at the mean (−0.006; p < 0.05).The RIF results show that a one standard deviation increase in adolescent neuroticism is associated with a decrease in the SF-6D at the 5th (−0.020; p < 0.01), 10th (−0.016; p < 0.01), 25th (−0.010; p < 0.1) and 50th (−0.005; p < 0.05), quantiles. The magnitude of the association decreases to around zero at the higher end of the health-related quality of life distribution. Once again, the standard errors are greatest at the lowest end of the SF-6D distribution. These results suggest that adolescent neuroticism is negatively associated with health-related quality of life at age 50, and that this association is greatest at the lower end of the unconditional SF-6D distribution.

#### 4.2.2 Biomarkers

##### 4.2.2.1 Allostatic load

Table 4 presents RIF regression results for the association between adolescent non-cognitive skills and allostatic load at ages 44–45. Higher levels of adolescent conscientiousness are weakly associated with a decrease in allostatic load, however we only find this at the 25th – 40th quantiles (1.6–1.9 %; p < 0.05). These results suggest there is very little, if any, association between adolescent conscientiousness and physiological ‘wear and tear’ at ages 44–45.

**Table 4:** OLS and RIF estimates of the associations between adolescent non-cognitive skills and the allostatic load index.

		RIF					
	OLS	Q10	Q25	Q50	Q75	Q90	Q95
Conscientiousness	−0.006	−0.002	−0.016**	−0.005	0.005	0.005	0.016
	(0.007)	(0.007)	(0.007)	(0.009)	(0.010)	(0.015)	(0.019)

Agreeableness	−0.010	0.002	0.002	−0.011	−0.015	−0.014	−0.053*
	(0.009)	(0.008)	(0.008)	(0.009)	(0.013)	(0.018)	(0.028)

Neuroticism	0.020***	0.008	0.011	0.015*	0.022**	0.031*	0.069***
	(0.007)	(0.009)	(0.008)	(0.008)	(0.011)	(0.018)	(0.024)
Observations	3165	3165	3165	3165	3165	3165	3165

Robust standard errors calculated from bootstrapping in parentheses: * p < 0.10, ** p < 0.05, *** p < 0.010.

**Notes:** the non-cognitive skills variables have been converted to their z statistic. The results can be interpreted as the effect of a one standard deviation increase in the non-cognitive skills. The outcome of interest is a log transformation of allostatic load and the results can be interpreted as a percentage change in the allostatic load index. Additional covariates include: age 11 test scores (general ability, reading, maths and copying design), child morbidity index, sex of the cohort member, mothers’ and fathers’ years of schooling, quadratic in mothers’ and fathers’ age in 1958, region of residence in 1958, parity of the mother in 1958, whether the mother or father had a long-term illness when the cohort member was a child, mothers’ and fathers’ birthplace, fathers’ social class and an indication of whether the parents had faced financial difficulties measured when the cohort member was 11.

**Fig. 3:**
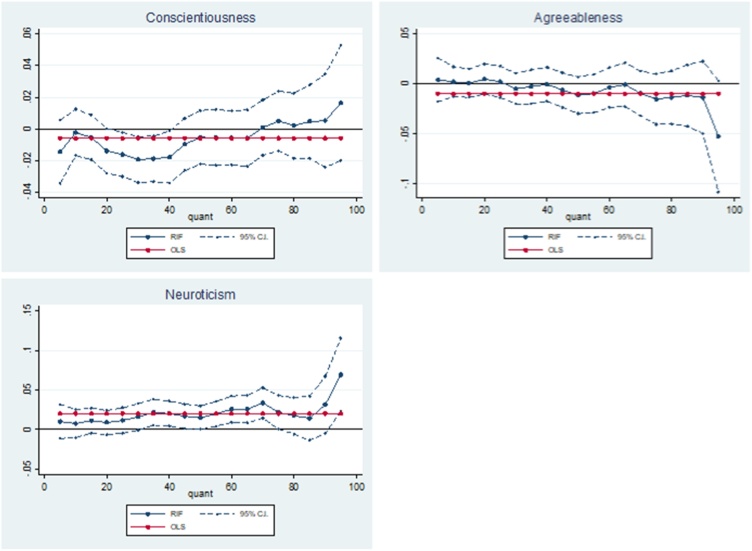
Adolescent non-cognitive skills – allostatic load relationship.

The results in Table 4 show that a one standard deviation increase in adolescent agreeableness is associated with 5.3 % (p < 0.1) decrease in allostatic load at the 95th quantile. The effect steadily decreases up the unconditional distribution, until the 95th quantile when it drops substantially (Fig. 3). These results suggest that higher levels of adolescent agreeableness are associated with lower levels of physiological ‘wear and tear’ for those at the upper extreme of the allostatic load distribution.

Lastly, we find that a one standard deviation increase in adolescent neuroticism is associated with an increase in allostatic load at the 50th (1.5 %; p < 0.1), 75th (2.2 %; p < 0.05), 90th (3.1 %; p < 0.1) and 95th (6.9 %; p < 0.01) quantiles. These results suggest that higher levels of adolescent neuroticism are associated with increased physiological ‘wear and tear’ and that these associations increase in magnitude at the high end of the distribution of physiological ‘wear and tear’.

##### 4.2.2.2 Cortisol t1-t2

A greater t1-t2 value (or greater difference) indicates a healthier stress response. Table 5 shows that a one standard deviation increase in adolescent conscientiousness is associated with an increase in cortisol t1-t2 at the 10th-75th quantiles, indicating a healthier stress response. An increase in adolescent conscientiousness is associated with an increase in cortisol t1-t2 by as much as 14.1 % (p < 0.01), which is found at the 10th quantile. The magnitude of this association decreases in quantiles representing lower levels of stress. However, at the 75th quantile higher levels of conscientiousness are still associated with a considerable increase in cortisol t1-t2 levels (4.6 %; p < 0.01). These results suggest that higher levels of adolescent conscientiousness are associated with decreased stress levels, and that this association is greatest for those with higher levels of stress.

**Table 5:** OLS and RIF estimates of the associations between adolescent non-cognitive skills and cortisol (t1-t2).

		RIF					
	OLS	Q5	Q10	Q25	Q50	Q75	Q90
Conscientiousness	0.062***	0.104	0.141***	0.060*	0.049**	0.046**	0.003
	(0.019)	(0.081)	(0.048)	(0.031)	(0.021)	(0.019)	(0.020)

Agreeableness	−0.039*	−0.096	−0.126***	−0.078**	−0.017	0.000	−0.002
	(0.020)	(0.083)	(0.053)	(0.034)	(0.025)	(0.021)	(0.020)

Neuroticism	−0.004	−0.053	−0.016	0.009	−0.014	0.004	−0.009
	(0.020)	(0.087)	(0.051)	(0.033)	(0.023)	(0.020)	(0.020)
Observations	2575	2575	2575	2575	2575	2575	2575

Robust standard errors calculated from bootstrapping in parentheses: * p < 0.10, ** p < 0.05, *** p < 0.010.

**Notes:** the non-cognitive skills variables have been converted to their z statistic. The results can be interpreted as the effect of a one standard deviation increase in the non-cognitive skills. The outcome of interest is a log transformation of cortisol t1-t2 and the changes can be interpreted as a percentage change in nmol/L. Additional covariates include: age 11 test scores (general ability, reading, maths and copying design), child morbidity index, sex of the cohort member, mothers’ and fathers’ years of schooling, quadratic in mothers’ and fathers’ age in 1958, region of residence in 1958, parity of the mother in 1958, whether the mother or father had a long-term illness when the cohort member was a child, mothers’ and fathers’ birthplace, fathers’ social class and an indication of whether the parents had faced financial difficulties measured when the cohort member was 11.

In comparison, a one standard deviation increase in adolescent agreeableness is associated with a decrease in cortisol t1-t2 at the 10th quantile (-12.6 %; p < 0.05) and the 25th quantile (-7.8 %; p < 0.05). As you move along the unconditional cortisol t1-t2 distribution, the magnitude of the association decreases to zero. Due to the inflated standard errors at the lower end of the distribution, significance is lost at the 5th quantile. These results suggest higher levels of adolescent agreeableness are associated with an increase in stress levels, and that this association is greatest for those with higher levels of stress.

**Fig. 4:**
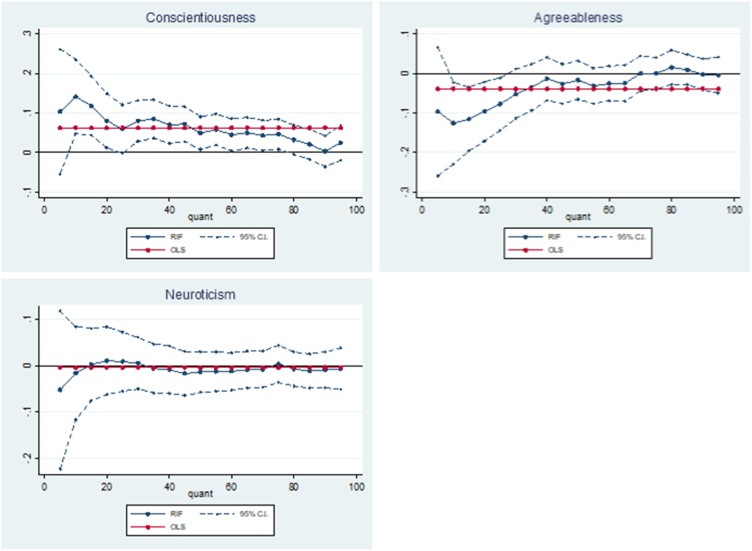
Adolescent non-cognitive skills – cortisol t1-t2 relationship.

Lastly, the results show that there is no significant association between adolescent neuroticism and cortisol t1-t2 at any point along its unconditional distribution. The effect sizes are much larger at the lower end of the distribution, and the effects switch from being positive to negative further up the distribution (Fig. 4).

##### 4.2.2.3 Triglyceride-HDL ratio

**Fig. 5:**
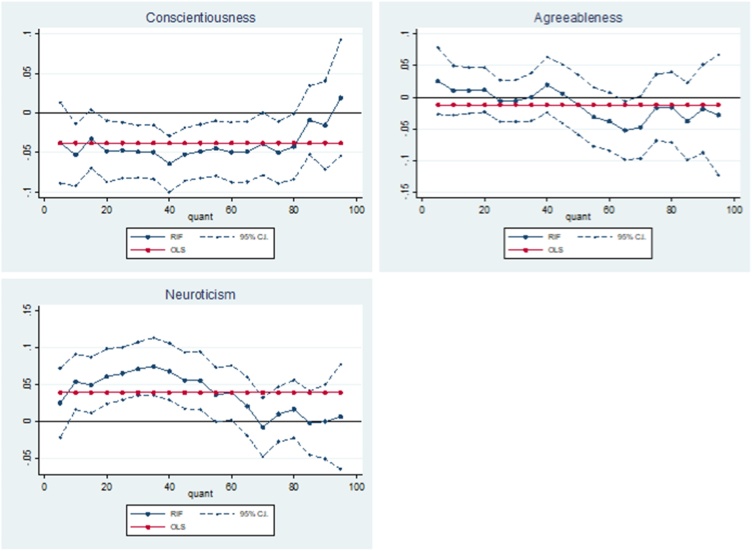
Adolescent non-cognitive skills – triglyceride-HDL ratio relationship.

The results in Table 6 show that a one standard deviation increase in adolescent conscientiousness is negatively associated with triglyceride-HDL ratio at the 10th-75th quantile, peaking at the 10th quantile (−5.3 %; p < 0.01). After the 75th quantile the magnitude of the negative association steadily decreases, becoming positive at the 95th quantile (Fig. 5). These results suggest that higher levels of adolescent conscientiousness are associated with a decrease in the risk of cardiovascular disease, and that this decreased risk is greatest for those who are in the low and medium risk groups for cardiovascular disease.

**Table 6:** OLS and RIF estimates of the associations between adolescent non-cognitive skills and the triglyceride-HDL ratio.

		RIF					
	OLS	Q10	Q25	Q50	Q75	Q90	Q95
Conscientiousness	−0.038***	−0.053***	−0.047***	−0.049***	−0.050**	−0.016	0.019
	(0.014)	(0.020)	(0.018)	(0.017)	(0.020)	(0.029)	(0.038)

Agreeableness	−0.012	0.011	−0.006	−0.012	−0.016	−0.018	−0.028
	(0.016)	(0.020)	(0.017)	(0.024)	(0.027)	(0.035)	(0.048)

Neuroticism	0.039***	0.054***	0.065***	0.055***	0.010	0.000	0.006
	(0.014)	(0.019)	(0.018)	(0.020)	(0.019)	(0.026)	(0.036)

Observations	3277	3277	3277	3277	3277	3277	3277

Robust standard errors calculated from bootstrapping in parentheses: * p < 0.10, ** p < 0.05, *** p < 0.010.

**Notes:** the non-cognitive skills variables have been converted to their z statistic. The results can be interpreted as a one standard deviation increase in the non-cognitive skills. The outcome of interest is a log transformation of triglyceride-HDL ratio and the results can be interpreted as a percentage change in the ratio. Additional covariates include: age 11 test scores (general ability, reading, maths and copying design), child morbidity index, sex of the cohort member, mothers’ and fathers’ years of schooling, quadratic in mothers’ and fathers’ age in 1958, region of residence in 1958, parity of the mother in 1958, whether the mother or father had a long-term illness when the cohort member was a child, mothers’ and fathers’ birthplace, fathers’ social class and an indication of whether the parents had faced financial difficulties measured when the cohort member was 11.

No significant association was found between adolescent agreeableness and triglyceride-HDL ratio along the unconditional distribution. However, the magnitude of the effects are greater at higher ends of the distribution.

The results show that a one standard deviation increase in adolescent neuroticism is positively associated with triglyceride-HDL ratio, which remains significant and relatively constant in magnitude at the 10th-50th quantile (5.4–6.5 %; p < 0.01). From the 75th quantile the magnitude of the association drops dramatically and loses signficance. These results suggest that higher levels of adolescent neuroticism are associated with an increase in the risk of cardiovascular disease, for those who are at low or medium risk of cardiovascular disease.

##### 4.2.2.4 C-reactive protein

**Fig. 6:**
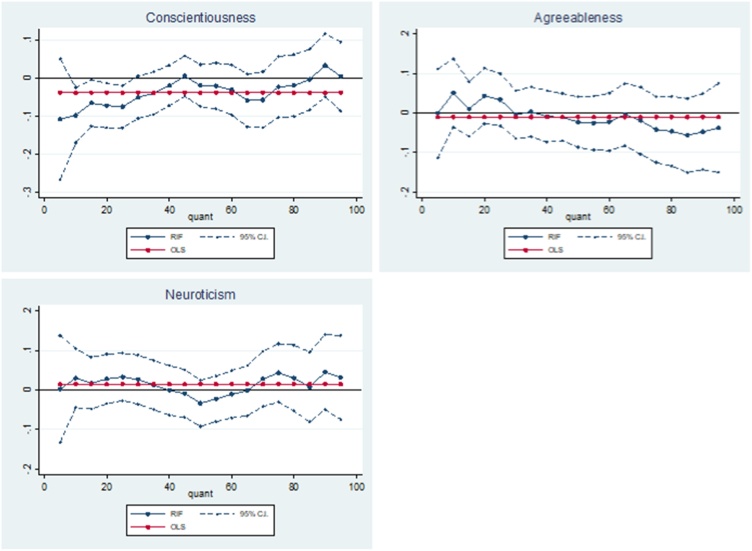
Adolescent non-cognitive skills – CRP relationship.

Table 7 shows that higher levels of adolescent conscientiousness are associated with lower levels of CRP at the 10th quantile (−9.8 %; p < 0.01) and the 25th quantile (−7.6 %; p < 0.01). The magnitude of this negative association decreases and becomes positive at the highest quantiles of the unconditional CRP distribution. The 95 % confidence interval stays relatively constant along the CRP distribution (Fig. 6). These results suggest adolescent conscientiousness is associated with a decreased risk of cardiovascular disease, however this association is only found for those low risk individuals.

**Table 7:** OLS and RIF estimates of the associations between adolescent non-cognitive skills and C-reactive protein (CRP).

		RIF					
	OLS	Q10	Q25	Q50	Q75	Q90	Q95
Conscientiousness	−0.039*	−0.098***	−0.076***	−0.020	−0.024	0.033	0.004
	(0.023)	(0.037)	(0.028)	(0.028)	(0.041)	(0.042)	(0.046)

Agreeableness	−0.010	0.051	0.034	−0.023	−0.042	−0.047	−0.038
	(0.028)	(0.044)	(0.034)	(0.033)	(0.042)	(0.049)	(0.058)

Neuroticism	0.015	0.030	0.033	−0.033	0.043	0.045	0.032
	(0.025)	(0.038)	(0.031)	(0.030)	(0.038)	(0.048)	(0.054)

Observations	3252	3252	3252	3252	3252	3252	3252

Robust standard errors calculated from bootstrapping in parentheses: * p < 0.10, ** p < 0.05, *** p < 0.010.

**Notes:** the non-cognitive skills variables have been converted to their z statistic. The results can be interpreted as a one standard deviation increase in the non-cognitive skills. The outcome of interest is a log transformation of CRP and the results can be interpreted as a percentage change in mg/L. Additional covariates include: age 11 test scores (general ability, reading, maths and copying design), child morbidity index, sex of the cohort member, mothers’ and fathers’ years of schooling, quadratic in mothers’ and fathers’ age in 1958, region of residence in 1958, parity of the mother in 1958, whether the mother or father had a long-term illness when the cohort member was a child, mothers’ and fathers’ birthplace, fathers’ social class and an indication of whether the parents had faced financial difficulties measured when the cohort member was 11.

No significant association was found between adolescent agreeableness and CRP at any point along the unconditional distribution of CRP. Similarly, no association was found between adolescent neuroticism and CRP in the RIF regressions.

### 4.3 Robustness checks

#### 4.3.1 Omitted variable bias

**Fig. 7:**
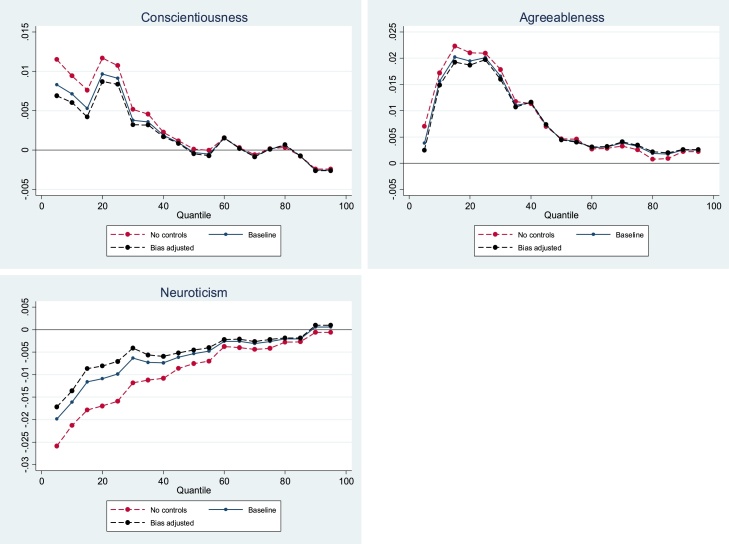
Adolescent non-cognitive skills – SF-6D omitted variable bias adjusted relationship.

Fig. 7 presents the causal bounds relating to the relationship between non-cognitive skills and the SF-6D index. For adolescent agreeableness, Bias-adjusted coefficients are almost identical to the baseline estimates along the whole SF-6D distribution, resulting in extremely narrow causal bounds. Adjusted coefficients for adolescent conscientiousness and neuroticism are almost identical to baseline estimates at the middle and the upper end of the SF-6D distribution, although bounds are slightly wider in magnitude at the lower end. Overall, these results suggest the estimates of the relationship between adolescent non-cognitive skills and the SF-6D at age 50 are robust to omitted variable bias.

**Fig. A4:**
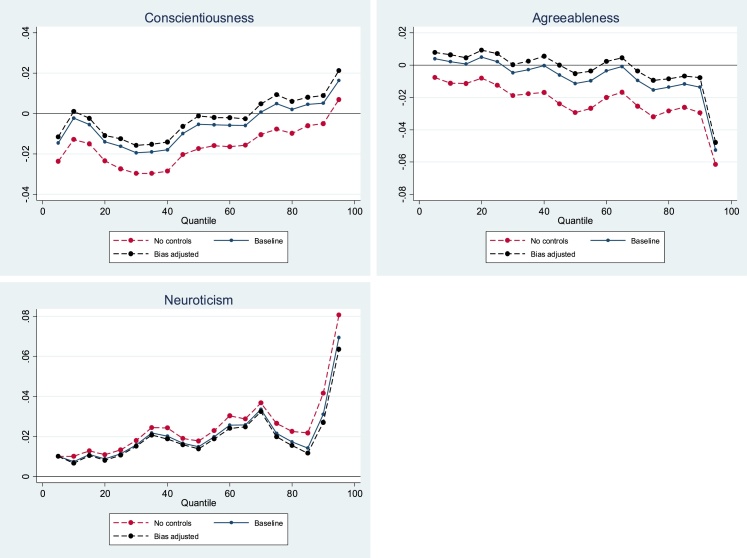
Adolescent non-cognitive skills – allostatic load omitted variable bias adjusted relationship.

Similarly, we find that all along the unconditional allostatic load distribution, bias-adjusted coefficients for adolescent neuroticism are almost identical to the baseline estimates (Appendix Fig. A4). For adolescent conscientiousness and agreeableness, causal bounds are wider but exclude zero at quantiles where statistically significant effects were detected in the main analysis.

**Fig. A5:**
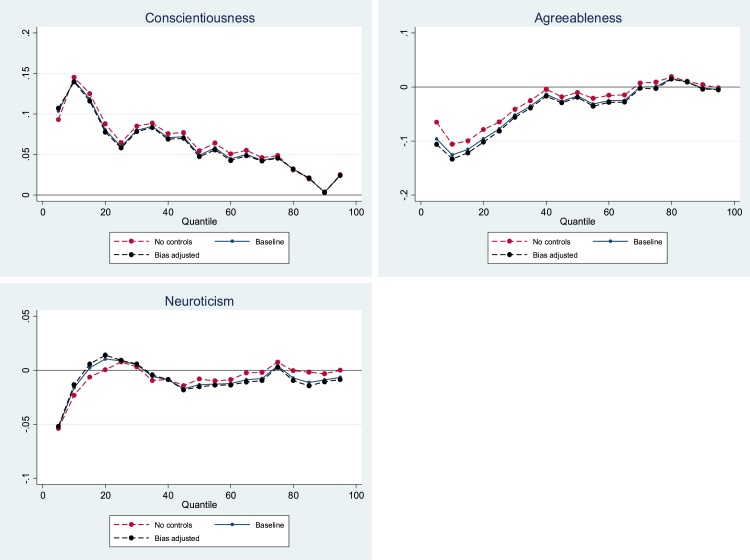
Adolescent non-cognitive skills – cortisol t1-t2 omitted variable bias adjusted relationship.

For cortisol t1-t2, bias-adjusted coefficients for all non-cognitive skills are almost identical to baseline estimates across the whole unconditional cortisol t1-t2 distribution (Appendix Figure A5).

**Fig. A6:**
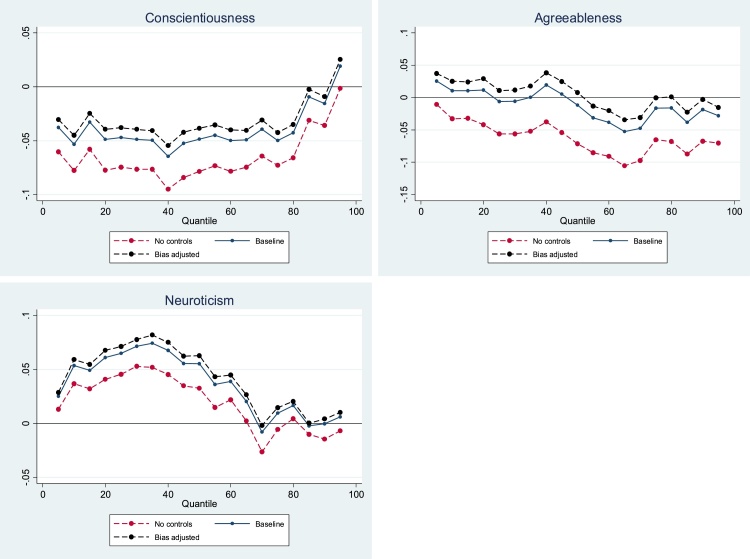
Adolescent non-cognitive skills – triglyceride-HDL ratio omitted variable bias adjusted relationship.

Appendix Fig. A6 presents results for the bias-adjusted relationships between adolescent non-cognitive skills and the triglyceride-HDL ratio. For adolescent conscientiousness, the bias-adjusted coefficients are slightly smaller in magnitude than the baseline estimates at all quantiles of the triglyceride-HDL ratio distribution. However, negative associations between adolescent conscientiousness and triglyceride-HDL ratio at the 5th-75th quantiles remain when adjusting for omitted variable bias. For adolescent agreeableness, bias-adjusted coefficients are a similar distance for our estimates along the whole unconditional triglyceride-HDL ratio distribution. Bias-adjusted coefficients for adolescent neuroticism are slightly larger than the baseline estimates, along the whole unconditional triglyceride-HDL ratio distribution (Figure A6).

**Fig. A7:**
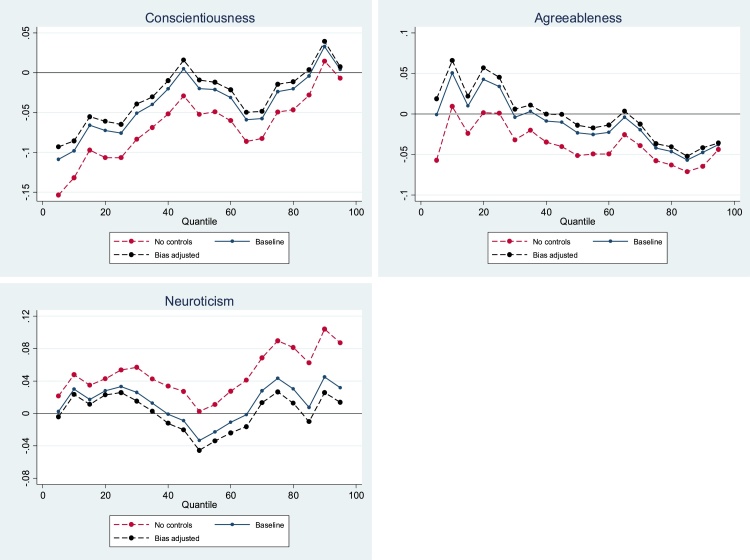
Adolescent non-cognitive skills – CRP omitted variable bias adjusted relationship.

For C-reactive protein, bias-adjusted association between adolescent conscientiousness are closest to our estimates at the upper and lower end of the CRP distribution (Appendix Fig. A7). For bias-adjusted associations relating to adolescent agreeableness are closest to the baseline estimates at the upper end of the unconditional CRP distribution. However, the results for adolescent neuroticism show that the bias-adjusted associations are closest to the baseline estimates at the lower end of the CRP distribution. Our bias adjusted CRP results do not change the conclusions we made in our original analysis.

Overall, these results suggest that omitted variable bias is unlikely to be affecting our results considerably.

#### 4.3.2 Non-random attrition bias

Table 8 present marginal effects from the propensity score model for the SF-6D estimation sample. The probability of being in this estimation sample is increasing in adolescent agreeableness, the general ability test score at age 11 and fathers’ years of schooling, and decreasing in adolescent neuroticism, being male, parity of the mother in 1958, fathers’ country of birth being outside of the UK and having a father whose social class is unskilled compared to professional. Results from propensity score models for the other estimation samples are similar to this, although for some region of residence in 1958 is also a significant predictor (Appendix Table A2, Table A3, Table A4, Table A5).

**Table 8:** Propensity score model – SF-6D sample.

Conscientiousness	0.006	Mothers’ birthplace	
	(0.004)	Wales	0.004
Agreeableness	0.016***		(0.041)
	(0.005)	Scotland	−0.016
Neuroticism	−0.016***		(0.049)
	(0.004)	Northern Ireland	0.060
Test scores (age 11)			(0.081)
General ability	0.004***	Ireland	−0.059
	(0.001)		(0.047)
Reading	0.000	Outside of the UK and Ireland	−0.014
	(0.002)		(0.036)
Maths	−0.001	Fathers’ birthplace	
	(0.001)	Wales	0.009
Copying design	−0.003		(0.039)
	(0.005)	Scotland	0.001
Child Morbidity Index (age 11)	−0.002		(0.044)
	(0.005)	Northern Ireland	−0.016
Financial Difficulties (age 11)	−0.002		(0.069)
	(0.023)	Ireland	−0.051
Male	−0.050***		(0.042)
	(0.013)	Outside of the UK and Ireland	−0.057*
Mothers' Age in 1958	−0.003		(0.034)
	(0.011)	Mother has a diagnosed illness	−0.042
Fathers' Age in 1958	0.008		(0.026)
	(0.008)	Fathers' has a diagnosed illness	−0.001
			(0.024)
Parity of the Mother in 1958	−0.009*	Region of residence 1958	
	(0.005)	North	−0.022
Mothers' Years of Schooling	−0.008		(0.025)
	(0.005)	North West	−0.029
Fathers' Years of Schooling	0.008*		(0.022)
	(0.005)	Riding	0.007
Fathers’ social class			(0.025)
II (Managerial and Technical)	−0.017	North Midlands	0.028
	(0.036)		(0.025)
III (Skilled – Non-Manual)	−0.046	Midlands	−0.020
	(0.039)		(0.023)
III (Skilled – Manual)	−0.044	East	0.041
	(0.036)		(0.025)
IV (Partly Skilled)	−0.030	South	0.009
	(0.040)		(0.028)
V (Unskilled)	−0.080*	South West	0.008
	(0.042)		(0.027)
		Wales	−0.015
			(0.046)

Standard errors in parentheses: * p < 0.10, ** p < 0.05, *** p < 0.010. N = 6,106.

**Fig. A8:**
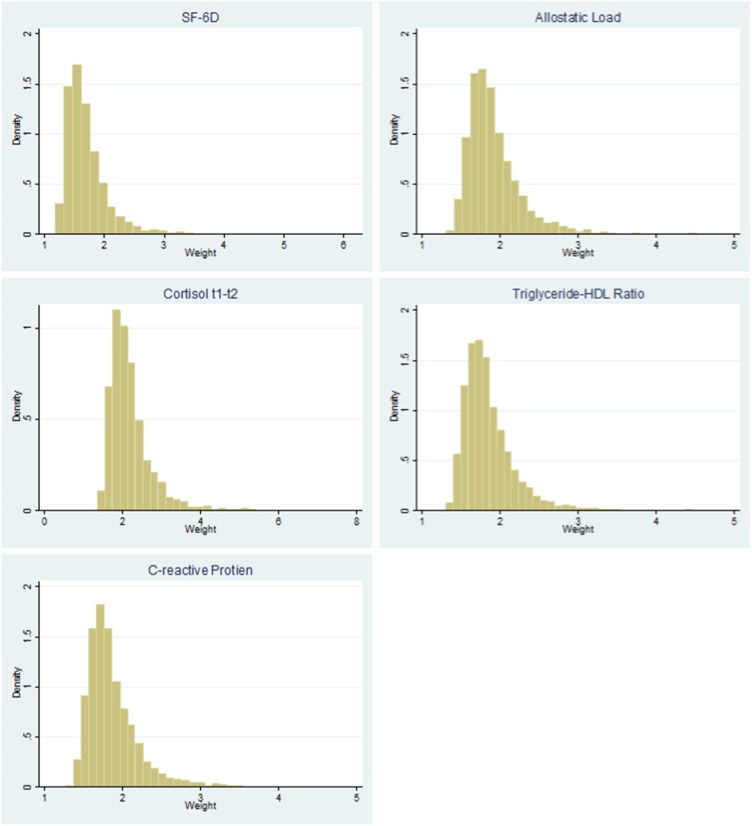
Distributions of inverse probability weights.

When applying IPW we find no extreme weights (Appendix Fig. A8), and following weighting characteristics of the estimation samples are now representative of the original sample (Appendix Table A6).

**Fig. A9:**
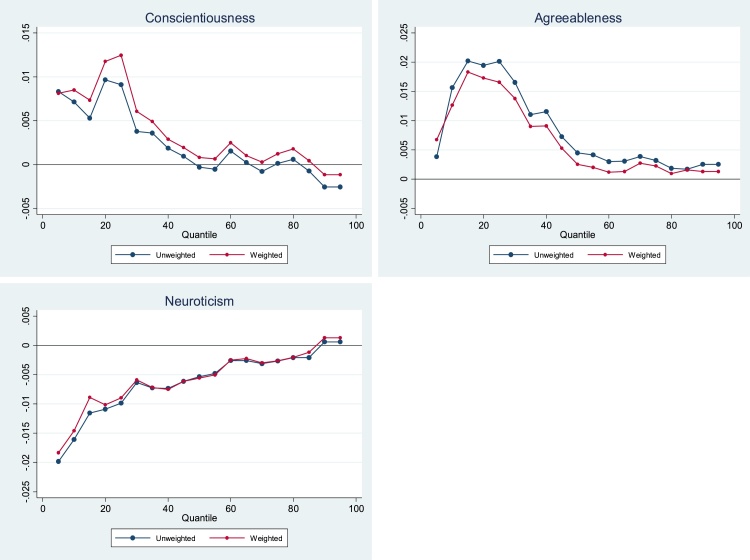
Weighted non-cognitive skills – SF-6D relationship.

**Fig. A10:**
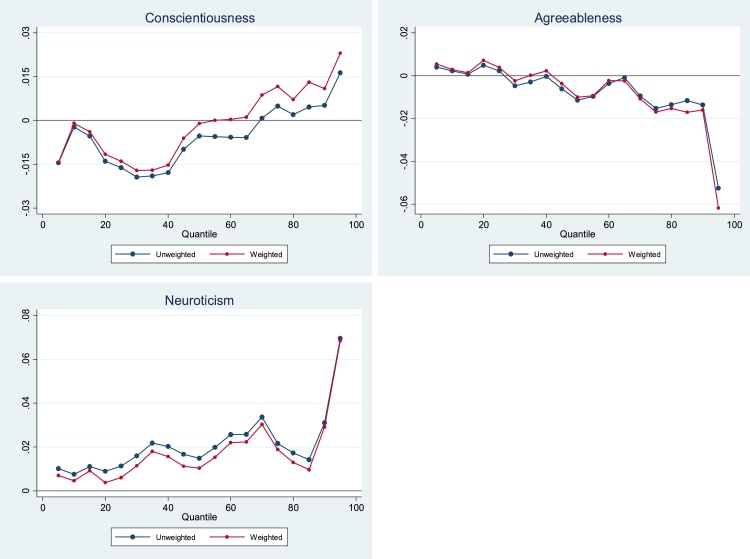
Weighted non-cognitive skills – Allostatic load relationship.

**Fig. A11:**
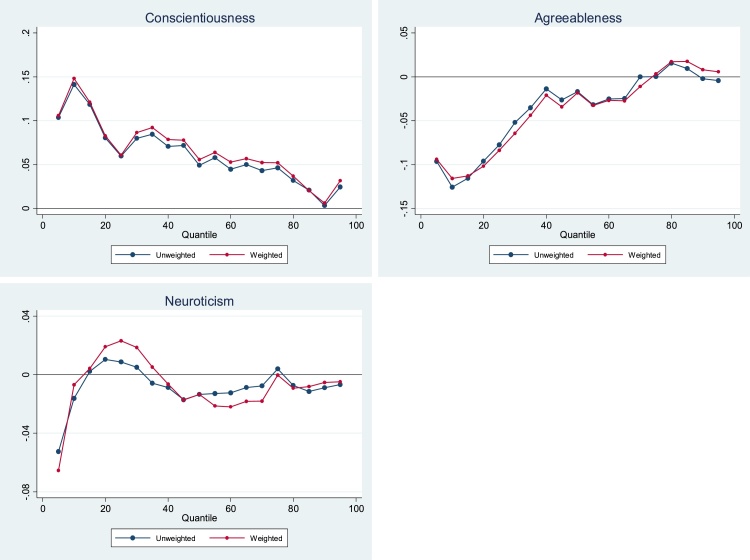
Weighted non-cognitive skills – cortisol t1-t2 relationship.

**Fig. A12:**
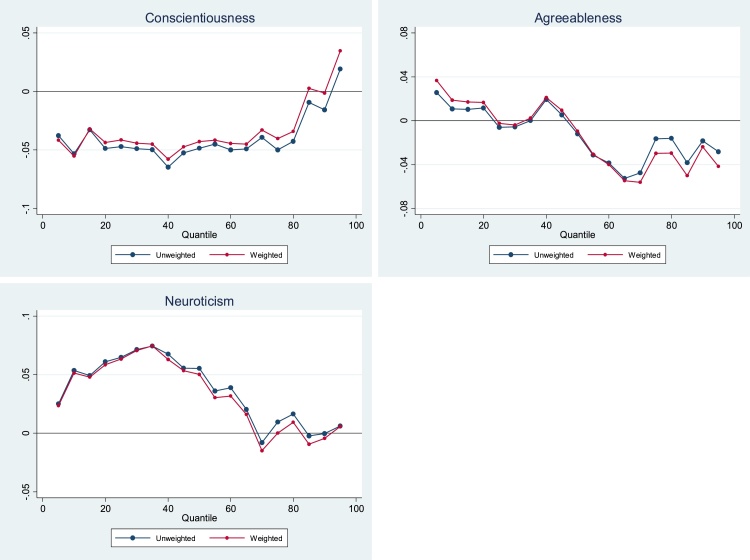
Weighted non-cognitive skills – triglyceride-HDL ratio relationship.

Appendix Fig. A9, Fig. A10, Fig. A11, Fig. A12 show estimates using IPW. In general, the results are relatively unaffected by weighting. For all of health outcomes, magnitudes change very slightly or not at all; and in some cases, the relationship becomes more pronounced. We find for all health outcomes, the pattern of the associations across the quantiles are unchanged, and our overall conclusions remain the same. These results suggest that, conditional on the MAR assumption being valid, attrition bias is unlikely to be affecting our results greatly.

## 5 Discussion and conclusion

We examined the association between adolescent non-cognitive skills and later-life health. In contrast to the previous literature in this area, we use a multidimensional definition of adolescent non-cognitive skills. Furthermore, we utilise both subjective and objective measures of health and study the association along the whole health distribution.

We use OLS and RIF regressions to study the relationship between adolescent non-cognitive skills and the unconditional distribution of later-life health. We use one measure of health-related quality of life: the SF-6D utility index. We also study four biomarkers as objective health measures: allostatic load, cortisol t1-t2, triglyceride-HDL ratio and CRP.

We found that adolescent conscientiousness is associated with a healthier (greater) stress response at the lower end of the stress response distribution, which indicates higher stress levels. We also found that higher adolescent conscientiousness is associated with a lower risk of cardiovascular disease and that the magnitude of this association is greater at the lower overall risk levels.

The RIF regression results suggest that adolescent agreeableness is associated with higher levels of health-related quality of life at the lower end of the distribution, indicating worse health-related quality of life. We also find higher adolescent agreeableness is associated with lower levels of physiological ‘wear and tear’. However, this is only true at the very extreme of the allostatic load distribution, for those with very high levels of physiological ‘wear and tear’. We also found that adolescent agreeableness is negatively associated with your ability to cope with stress, and the magnitude of this association is greatest for those individuals with higher levels of stress.

Lastly, we found that higher adolescent neuroticism was associated with poorer health-related quality of life at the lower end of the distribution, indicating worse health-related quality of life. We also found adolescent neuroticism was associated with an increase in physiological ‘wear and tear’, and that this association increased as you moved from low to high levels of physiological ‘wear and tear’. Lastly, we found that higher adolescent neuroticism was associated with an increased risk of cardiovascular disease. However, this increase was greatest for those at the low- to mid-risk level.

The fact we find most of our associations vary along the unconditional distribution of health, is evidence of how OLS masks important differences in the relationship between adolescent non-cognitive skills and later-life health across the health distribution.

Overall, we find our results are likely to be robust to omitted variable bias and non-random attrition bias. In some cases we found bias-adjusted coefficients calculated using methods in Oster (2019) to be almost identical to baseline estimates, suggesting in these cases that our estimates are close to the true causal effect.

### 5.1 Comparisons to previous literature

Although evidence exists that shows higher levels of neuroticism are associated with lower self-reported health (Jorm et al., 1993; de Jonge and Slaets, 2005d; Turiano et al., 2012; Magee et al., 2013), this is the first study to look at adolescent neuroticism and show that this association varies along the health distribution. In comparison, evidence suggests that higher levels of adult conscientiousness and agreeableness are associated with higher self-reported health (Löckenhoff et al., 2011; Turiano et al., 2012). We find the same relationship for adolescent agreeableness; however, we find no association between adolescent conscientiousness and the SF-6D. This may be because the existing evidence uses contemporaneous measures of non-cognitive skills rather than measures recorded at age 16.

The lack of a significant association between adolescent conscientiousness and the SF-6D is also inconsistent with the previous evidence that suggests self-control, measured in childhood, is positively associated with self-reported health in adulthood (Cutler and Lleras-Muney, 2010; Kaestner and Callison, 2011). However, self-control is only one facet of conscientiousness and therefore the facets included in our measure may not have the same relationship with self-reported health. Furthermore, the difference in results may be due to the fact that our self-reported health measure is utility weighted and is a measure of health-related quality of life, not solely self-reported health.

Similarly to Hampson et al. (2013) we find a negative association between conscientiousness and our metabolic biomarker, the triglyceride-HDL ratio, in both our OLS and RIF regression analysis. We do not find an association between conscientiousness and physiological ‘wear and tear’, at the mean. However, consistent with Hampson et al. (2013) we find an association in the same direction at the 25th–40th quantiles of the unconditional allostatic load distribution. As there is no consensus on the biomarkers that should be used to create a measure of allostatic load, these measures vary greatly between studies (Johnson et al., 2017). The difference in the biomarkers used to create measures of physiological ‘wear and tear’ between our studies may explain why we did not find an association at the mean.

Similarly to the psychology literature on adult conscientiousness, we find adolescent conscientiousness is positively associated with your ability to cope with stress (Soliemanifar et al., 2018), and negatively associated with biomarkers for cardiovascular risk (Sutin et al., 2010b; Luchetti et al., 2014; Elliot et al., 2017; Wagner et al., 2019). Our results suggest that the relationship between conscientiousness and health may stay relatively constant over the life course. We build on this evidence by showing that the magnitude of these associations varies substantially along the unconditional distribution of the biomarkers, suggesting a lot of information about the true relationship is missed by the association at the mean.

Similarly to findings from this study, there is some evidence to suggest that adult agreeableness is associated with lower allostatic load (Christensen et al., 2019). However, we only found an association at the very extreme of the unconditional allostatic load distribution, which indicates high physiological ‘wear and tear’. Again, this may be due to the differences in biomarkers used to create our allostatic load measures (Johnson et al., 2017). Previous studies have also shown that agreeableness is positively associated with cortisol levels. We find the same relationship between agreeableness and stress at the mean. However, we build on this evidence by showing that the association is greatest for those individuals who have higher levels of stress.

Lastly, the current evidence on adult neuroticism is in line with our results that suggests higher adolescent neuroticism is associated with higher allostatic load (Stephan et al., 2016), and higher levels of the triglyceride-HDL ratio (Sutin et al., 2010a; Roh et al., 2014). We build on this evidence by showing how the association varies along their unconditional distributions. Unlike the evidence on adult neuroticism, we do not find an association between adolescent neuroticism and cortisol (Soliemanifar et al., 2018). This may suggest the relationship does not stay constant over the life course.

As we are the first study to look at the association between non-cognitive skills and health over the unconditional distribution of health, we can only comment on whether the pattern of the associations along the distributions seems intuitive. In our SF-6D, allostatic load and cortisol t1-t2 analyses we find that the magnitude of the association is greatest at the extreme of the distribution that indicates worse health or a higher risk level of disease. However, this is in contrast to the triglyceride-HDL ratio and CRP analyses, where the associations are greater at the lower end of the risk distributions. One explanation for this is that the mediators in these relationships may explain where in the distributions associations are greatest. CRP and the triglyceride-HDL ratio are both biomarkers for cardiovascular disease, therefore these may have different mediators to the SF-6D, allostatic load and cortisol that are measuring general health and stress.

### 5.2 Limitations

A limitation of this study is that the biomarkers were collected at ages 44–45, which may be too early for many health issues to have manifested. The biomarkers act as an indication of the risk of disease, rather than their presence.

Secondly, substantial sample drop-out between adolescence and adulthood increases the risk of non-random attrition bias. However, although we find that individuals that remain in our estimation sample differ significantly from lost observations on cognitive ability, parental socioeconomic status and conscientiousness, results are relatively unchanged when results are re-estimated using inverse probability weighting. However, IPW only corrects for attrition bias if the MAR assumption holds, and there is always a possibility that some predictors of missingness are not included in the propensity score model. Furthermore, our study is limited by the fact that we are only able to find associations and we cannot claim causality. However, results are robust to adjusting coefficients for omitted variable bias using the Oster (2019) method, increasing the plausibility of a causal interpretation of results.

Lastly, we are limited by the fact that we do not know the mechanisms in the relationship between adolescent non-cognitive skills and adult health. As our non-cognitive skills variables are recorded at age 16 and the biomarkers aren’t measured till ages 44–45, there is clear scope for a mediation analysis to understand the direct and indirect pathways in these relationships. In particular, the role that lifestyle behaviours, including smoking, alcohol consumption, diet and exercise, play in the relationship between adolescent non-cognitive skills and adult health. A better understanding of these mediating pathways will guide the sort of public policies needed alongside those needed to improve non-cognitive skills.

### 5.3 Policy implications

Our results suggest that the level of non-cognitive skills an individual has at age 16 plays an important role in determining their later-life health outcomes. When we look at our results alongside studies on the association between adult non-cognitive skills and health, we find some evidence that this relationship stays relatively constant over the life course. However, there is growing evidence to suggest that non-cognitive skills do not stay constant over the life course, and are malleable (Heckman and Kautz, 2012). Therefore, our findings build on the evidence in support of interventions to help improve these skills; such as the Perry Program and the Abecedarian Project (Conti et al., 2016; Schweinhart and Weikart, 1981). In particular, our analysis of unobservables suggests the need for policy to improve conscientiousness and reduce neuroticism, to help improve adult health. We are unable to comment on agreeableness as our results are inconclusive for whether it is good or bad for adult health. These results help broaden our understanding of the importance of non-cognitive skills in determining later-life outcomes. As a result, we will be better able to evaluate such interventions.

This implication is becoming increasingly important as policy moves towards more complex public health interventions (Schonert-Reichl et al., 2012; Baba et al., 2017). Evaluations of these interventions are beginning to recognise the role of non-cognitive skills as an instrument through which health can be improved (Craig et al., 2006; Deidda et al., 2018). The evidence presented here emphasises the importance of their inclusion in such evaluations.

More specifically, our analysis of the biomarkers suggest that adolescent non-cognitive skills are associated not just with general health, but also with individual health conditions. For example, we find adolescent conscientiousness is negatively associated with both biomarkers for cardiovascular risk. In comparison, we find adolescent neuroticism is associated with higher levels of the triglyceride-HDL ratio. These results suggest that improving adolescent conscientiousness and reducing adolescent neuroticism could reduce the risk of cardiovascular disease beginning to emerge at ages 44–45.

## Funding

Medical Research Council Doctoral Training Programme. Grant number: MR/N013751/1.

## Declaration of Competing Interest

None.

## Figures and Tables

**Fig. A13:**
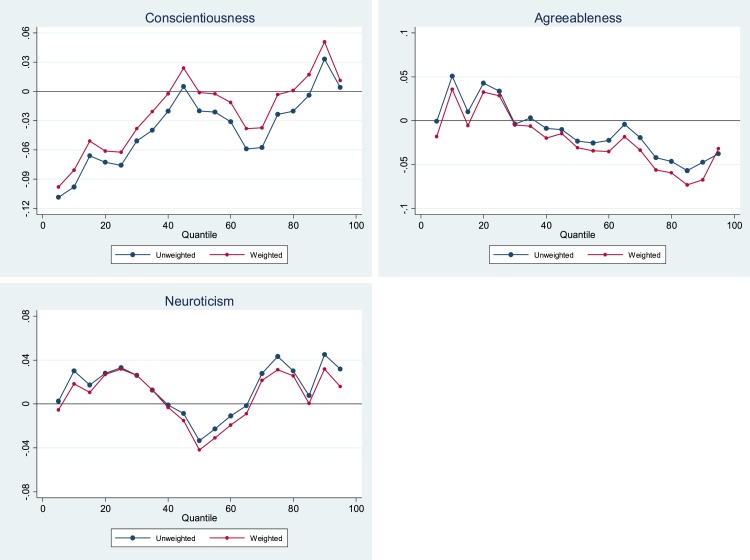
Weighted non-cognitive skills – C-reactive protein relationship.

**Table A1:** Description of biomarker.

Biomarker	What does it measure?
Salivary cortisol (morning)	Elevated cortisol levels have been shown to predict stress, depression and Cushing’s syndrome (Carroll et al., 2016; Hellhammer et al., 2009; Owens et al., 2014).
Salivary cortisol (morning – afternoon)	A slower decrease in cortisol levels through out the day (flatter diurnal cortisol slopes) is an indicator of high levels of stress and is associated with health conditions such as depression, fatigue, cardiovascular disease and type II diabetes (Adam et al., 2017; Bower et al., 2005; Doane et al., 2013; Hackett et al., 2016; Kumari et al., 2009; Matthews et al., 2006).
Insulin-life growth factor-1 (IGF-1)	High serum IGF-1 is associated with increased risk of several common cancers, including breast and lung cancer. Lower concentrations or circulating IGF-1 have been associated with osteoporosis (Garg et al., 1997).
C-reactive protein (CRP)	Mild increases in CRP levels (2–10 mg/L) is an indicator of metabolic inflammation and is associated with chronic diseases such as cardiovascular disease and type II diabetes (Brenner et al., 2014; Yale et al., 2016).
Fibrinogen	Hypofibrinogenemia is characterised by fibrinogen levels lower than 1.5 g/l and leaves you at risk of bleeding after surgery (Neerman-Arbez and Casini, 2018). High levels (>4 g/L) of fibrinogen has been shown to act as a predictor of cardiovascular disease (Tousoulis et al., 2011).
Immunoglobulin E (IgE)	IgE is an important regulator of allergic reactions (Helby et al., 2015). High levels of IgE are associated with cardiovascular disease and the incidence of acute myocardial infraction (Kounis and Hahalis, 2016).
High-density lipoprotein (HDL)	HDL is a type of cholesterol, and is commonly known as the “good” cholesterol as high levels are associated with reduced levels of cardiovascular disease (Castelli et al., 1986; Fisher et al., 2012).
Low-density lipoprotein (LDL)	High levels of LDL (>160 nmol/L) predicts cardiovascular disease (Boden, 2000).
Triglyceride	High levels of triglyceride (>200 mg/dL) shown in patients with incident ischemic stroke, and is associated with increased cardiovascular risk (Miller et al., 2011; Upadhyay, 2015; Wedro, 2018).
Glycated Haemoglobin (HbA1c)	HbA1c levels between 5.7 % and 6.4 % indicate that you have a high chance of getting diabetes. Levels of 6.5 % or higher indicate the presence of diabetes (Lyons and Basu, 2012)
Systolic blood pressure	Systolic blood pressure greater than or equal to 130 mmHg is a diagnosis of hypertension (Shere et al., 2017).
Diastolic blood pressure	Diastolic blood pressure greater than or equal to 80 mmHg is a diagnosis of hypertension (Shere et al., 2017).
Heart rate	Heart rate is a determinant of myocardial oxygen demand, coronary blood flow, and myocardial performance and is central to the adaptation of cardiac output to metabolic needs. Increased heart rate is known to predict adverse outcome in the general population and in patients with chronic heart failure (Shaaya et al., 2017).
Expiratory flow	The peak expiratory flow test is a common test that helps to diagnose and check lung problems, such as asthma and chronic obstructive pulmonary disease (So et al., 2016).

**Table A2:** Propensity score model – Allostatic load sample.

Conscientiousness	−0.001	Mothers’ birthplace	
	(0.004)	Wales	0.026
Agreeableness	0.015***		(0.042)
	(0.005)	Scotland	0.011
Neuroticism	−0.012***		(0.050)
	(0.004)	Northern Ireland	−0.012
Test scores (age 11)			(0.083)
General ability	0.003***	Ireland	−0.068
	(0.001)		(0.049)
Reading	−0.003	Outside of the UK and Ireland	−0.053
	(0.002)		(0.037)
Maths	0.000	Fathers’ birthplace	
	(0.001)	Wales	−0.001
Copying design	0.006		(0.040)
	(0.004)	Scotland	−0.049
Child Morbidity Index (age 11)	−0.003		(0.045)
	(0.005)	Northern Ireland	0.117
Financial Difficulties (age 11)	−0.009		(0.073)
	(0.024)	Ireland	−0.001
Male	−0.009		(0.044)
	(0.013)	Outside of the UK and Ireland	−0.046
Mothers' Age in 1958	0.000		(0.036)
	(0.012)	Mother has a diagnosed illness	−0.043
Fathers' Age in 1958	0.014		(0.027)
	(0.009)	Fathers' has a diagnosed illness	0.016
			(0.025)
Parity of the Mother in 1958	0.002	Region of residence 1958	
	(0.006)	North	0.049*
Mothers' Years of Schooling	−0.005		(0.026)
	(0.005)	North West	−0.043*
Fathers' Years of Schooling	0.009*		(0.023)
	(0.005)	Riding	0.046*
Fathers’ social class			(0.025)
II (Managerial and Technical)	0.027	North Midlands	0.072***
	(0.035)		(0.026)
III (Skilled – Non-Manual)	0.019	Midlands	0.006
	(0.038)		(0.024)
III (Skilled – Manual)	0.029	East	0.031
	(0.035)		(0.026)
IV (Partly Skilled)	0.033	South	0.023
	(0.039)		(0.029)
V (Unskilled)	−0.058	South West	0.002
	(0.042)		(0.028)
		Wales	−0.055
			(0.048)

Standard errors in parentheses: * p < 0.10, ** p < 0.05, *** p < 0.010. N = 6,106.

**Table A3:** Propensity score model – cortisol t1-t2 sample.

Conscientiousness	0.011***	Mothers’ birthplace	
	(0.004)	Wales	0.031
Agreeableness	0.015***		(0.042)
	(0.005)	Scotland	−0.091*
Neuroticism	−0.017***		(0.052)
	(0.004)	Northern Ireland	−0.063
Test scores (age 11)			(0.085)
General ability	0.004***	Ireland	−0.062
	(0.001)		(0.050)
Reading	−0.004***	Outside of the UK and Ireland	0.014
	(0.002)		(0.037)
Maths	0.001	Fathers’ birthplace	
	(0.001)	Wales	−0.050
Copying design	0.007		(0.040)
	(0.005)	Scotland	−0.038
Child Morbidity Index (age 11)	−0.003		(0.046)
	(0.005)	Northern Ireland	0.038
Financial Difficulties (age 11)	−0.021		(0.071)
	(0.025)	Ireland	0.067
Male	−0.018		(0.045)
	(0.013)	Outside of the UK and Ireland	−0.006
Mothers' Age in 1958	−0.016		(0.035)
	(0.012)	Mother has a diagnosed illness	−0.018
Fathers' Age in 1958	0.018**		(0.028)
	(0.009)	Fathers' has a diagnosed illness	−0.003
			(0.025)
Parity of the Mother in 1958	0.006	Region of residence 1958	
	(0.006)	North	0.037
Mothers' Years of Schooling	−0.011**		(0.026)
	(0.005)	North West	−0.034
Fathers' Years of Schooling	0.003		(0.023)
	(0.005)	Riding	0.045*
Fathers’ social class			(0.025)
II (Managerial and Technical)	0.036	North Midlands	0.068***
	(0.035)		(0.025)
III (Skilled – Non-Manual)	−0.031	Midlands	0.022
	(0.038)		(0.024)
III (Skilled – Manual)	−0.019	East	0.045*
	(0.035)		(0.025)
IV (Partly Skilled)	−0.035	South	0.008
	(0.039)		(0.029)
V (Unskilled)	−0.065	South West	0.035
	(0.042)		(0.027)
		Wales	0.014
			(0.047)

Standard errors in parentheses: * p < 0.10, ** p < 0.05, *** p < 0.010. N = 6,106.

**Table A4:** Propensity score model – triglyceride-HDL ratio sample.

Conscientiousness	0.000	Mothers’ birthplace	
	(0.004)	Wales	0.010
Agreeableness	0.016***		(0.042)
	(0.005)	Scotland	−0.002
Neuroticism	−0.012***		(0.050)
	(0.004)	Northern Ireland	0.002
Test scores (age 11)			(0.083)
General ability	0.004***	Ireland	−0.062
	(0.001)		(0.049)
Reading	−0.003**	Outside of the UK and Ireland	−0.035
	(0.002)		(0.037)
Maths	0.000	Fathers’ birthplace	
	(0.001)	Wales	0.004
Copying design	0.007		(0.040)
	(0.005)	Scotland	−0.063
Child Morbidity Index (age 11)	−0.001		(0.045)
	(0.005)	Northern Ireland	0.097
Financial Difficulties (age 11)	−0.017		(0.073)
	(0.024)	Ireland	0.008
Male	−0.007		(0.044)
	(0.013)	Outside of the UK and Ireland	−0.047
Mothers' Age in 1958	−0.001		(0.035)
	(0.012)	Mother has a diagnosed illness	−0.041
Fathers' Age in 1958	0.012		(0.027)
	(0.009)	Fathers' has a diagnosed illness	0.018
			(0.025)
Parity of the Mother in 1958	0.001	Region of residence 1958	
	(0.006)	North	0.053**
Mothers' Years of Schooling	−0.005		(0.026)
	(0.005)	North West	−0.037
Fathers' Years of Schooling	0.008*		(0.023)
	(0.005)	Riding	0.048*
Fathers’ social class			(0.025)
II (Managerial and Technical)	0.037	North Midlands	0.070***
	(0.035)		(0.026)
III (Skilled – Non-Manual)	0.024	Midlands	0.016
	(0.038)		(0.024)
III (Skilled – Manual)	0.040	East	0.044*
	(0.035)		(0.026)
IV (Partly Skilled)	0.036	South	0.026
	(0.039)		(0.029)
V (Unskilled)	−0.041	South West	0.011
	(0.042)		(0.028)
		Wales	−0.045
			(0.047)

Standard errors in parentheses: * p < 0.10, ** p < 0.05, *** p < 0.010. N = 6,106.

**Table A5:** Propensity score model – C-reactive protein sample.

Conscientiousness	−0.002	Mothers’ birthplace	
	(0.004)	Wales	0.042
Agreeableness	0.014***		(0.042)
	(0.005)	Scotland	−0.003
Neuroticism	−0.013***		(0.050)
	(0.004)	Northern Ireland	0.008
Test scores (age 11)			(0.083)
General ability	0.003***	Ireland	−0.065
	(0.001)		(0.049)
Reading	−0.002	Outside of the UK and Ireland	−0.031
	(0.002)		(0.037)
Maths	0.000	Fathers’ birthplace	
	(0.001)	Wales	−0.009
Copying design	0.006		(0.040)
	(0.005)	Scotland	−0.047
Child Morbidity Index (age 11)	−0.005		(0.045)
	(0.005)	Northern Ireland	0.099
Financial Difficulties (age 11)	−0.001		(0.073)
	(0.024)	Ireland	−0.016
Male	−0.011		(0.044)
	(0.013)	Outside of the UK and Ireland	−0.053
Mothers' Age in 1958	0.001		(0.035)
	(0.012)	Mother has a diagnosed illness	−0.028
Fathers' Age in 1958	0.014		(0.027)
	(0.009)	Fathers' has a diagnosed illness	0.013
			(0.025)
Parity of the Mother in 1958	0.000	Region of residence 1958	
	(0.006)	North	0.049*
Mothers' Years of Schooling	−0.005		(0.026)
	(0.005)	North West	−0.035
Fathers' Years of Schooling	0.010**		(0.023)
	(0.005)	Riding	0.050**
Fathers’ social class			(0.025)
II (Managerial and Technical)	0.022	North Midlands	0.069***
	(0.035)		(0.026)
III (Skilled – Non-Manual)	0.008	Midlands	0.008
	(0.038)		(0.024)
III (Skilled – Manual)	0.031	East	0.025
	(0.035)		(0.026)
IV (Partly Skilled)	0.029	South	0.033
	(0.039)		(0.029)
V (Unskilled)	−0.058	South West	0.008
	(0.042)		(0.028)
		Wales	−0.050
			(0.047)

Standard errors in parentheses: * p < 0.10, ** p < 0.05, *** p < 0.010. N = 6,106.

**Table A6:** Mean covariates – baseline, weighted and total sample.

	Baseline estimation sample	Weighted sample	Total sample
Conscientiousness	6.45	6.34	6.35
Agreeableness	11.55	11.37	11.38
Neuroticism	1.07	1.21	1.22

Test scores			
General ability	47.29	45.02	44.93
Reading	17.28	16.63	16.57
Maths	18.88	17.71	17.66
Copying designs	8.50	8.44	8.44
Financial difficulties	0.07	0.08	0.08

Fathers’ social class			
I (Professional)	0.06	0.05	0.05
II	0.16	0.14	0.14
III	0.10	0.10	0.10
III	0.50	0.51	0.51
IV	0.11	0.12	0.12
V	0.07	0.08	0.08
Mothers’ years of schooling	15.05	14.99	14.99
Fathers’ years of schooling	15.10	14.99	14.99

**Notes**: SF-6D baseline estimation sample and weighted sample used.

## Appendix A

Fig. A13

## Footnotes

1 We chose not to include the biomarker for diabetes (HbA1c) as no individual in our sample had levels of the biomarker that indicated the presence of diabetes or prediabetes. We also found no significant results when carrying out our analysis on HbA1c.

2 Parental health measure is an indicator of whether the parent has been diagnosed with any illness. These include: unknown illness, dermatological, cardiovascular, respiratory, psychiatric, subnormal and educationally subnormal, urogenital, alimentary, locomotor, neurological, infections and special senses.

3 47% of the observations dropped had missing data on all of the controls. A large driver of the rest of the dropped observations was missing data for mothers’ and fathers’ birthplace.

4 Results at the 5^th^ quantile are only presented for those health outcomes for which the lower quantiles represent poorer health. Similarly, results at the 95^th^ quantile are only presented for those health outcomes for which the upper quantiles represent poorer health.
